# The risk of sudden cardiac arrest and ventricular arrhythmia with rosiglitazone versus pioglitazone: real-world evidence on thiazolidinedione safety

**DOI:** 10.1186/s12933-020-00999-5

**Published:** 2020-02-25

**Authors:** Charles E. Leonard, Colleen M. Brensinger, Ghadeer K. Dawwas, Rajat Deo, Warren B. Bilker, Samantha E. Soprano, Neil Dhopeshwarkar, James H. Flory, Zachary T. Bloomgarden, Joshua J. Gagne, Christina L. Aquilante, Stephen E. Kimmel, Sean Hennessy

**Affiliations:** 1 Center for Pharmacoepidemiology Research and Training, Department of Biostatistics, Epidemiology, and Informatics, Perelman School of Medicine, University of Pennsylvania, 423 Guardian Drive, Philadelphia, PA 19104 USA; 2 Division of Cardiovascular Medicine, Department of Medicine, Perelman School of Medicine, University of Pennsylvania, 3400 Spruce Street, Philadelphia, PA 19104 USA; 3 Endocrinology Service, Department of Subspecialty Medicine, Memorial Sloan Kettering Cancer Center, 1275 York Avenue, New York, NY 10065 USA; 4 Division of Endocrinology and Metabolism, Department of Medicine, Icahn School of Medicine at Mount Sinai, 35 East 85th Street, New York, NY 10028 USA; 5 Division of Pharmacoepidemiology and Pharmacoeconomics, Department of Medicine, Brigham and Women’s Hospital and Harvard Medical School, Harvard University, 1620 Tremont Street, Boston, MA 02120 USA; 6 Department of Pharmaceutical Sciences, Skaggs School of Pharmacy and Pharmaceutical Sciences, Anschutz Medical Campus, University of Colorado, 12850 E. Montview Boulevard, Aurora, CO 80045 USA; 7 Department of Systems Pharmacology and Translational Therapeutics, Perelman School of Medicine, University of Pennsylvania, Philadelphia, PA 19104 USA

**Keywords:** Thiazolidinediones, Type 2 diabetes mellitus, Sudden cardiac death, Cardiac arrhythmias, Cohort studies, Pharmacoepidemiology, Propensity score, Medicaid

## Abstract

**Background:**

The low cost of thiazolidinediones makes them a potentially valuable therapeutic option for the > 300 million economically disadvantaged persons worldwide with type 2 diabetes mellitus. Differential selectivity of thiazolidinediones for peroxisome proliferator-activated receptors in the myocardium may lead to disparate arrhythmogenic effects. We examined real-world effects of thiazolidinediones on outpatient-originating sudden cardiac arrest (SCA) and ventricular arrhythmia (VA).

**Methods:**

We conducted population-based high-dimensional propensity score-matched cohort studies in five Medicaid programs (California, Florida, New York, Ohio, Pennsylvania | 1999–2012) and a commercial health insurance plan (Optum Clinformatics | 2000–2016). We defined exposure based on incident rosiglitazone or pioglitazone dispensings; the latter served as an active comparator. We controlled for confounding by matching exposure groups on propensity score, informed by baseline covariates identified via a data adaptive approach. We ascertained SCA/VA outcomes precipitating hospital presentation using a validated, diagnosis-based algorithm. We generated marginal hazard ratios (HRs) via Cox proportional hazards regression that accounted for clustering within matched pairs. We prespecified Medicaid and Optum findings as primary and secondary, respectively; the latter served as a conceptual replication dataset.

**Results:**

The adjusted HR for SCA/VA among rosiglitazone (vs. pioglitazone) users was 0.91 (0.75–1.10) in Medicaid and 0.88 (0.61–1.28) in Optum. Among Medicaid but not Optum enrollees, we found treatment effect heterogeneity by sex (adjusted HRs = 0.71 [0.54–0.93] and 1.16 [0.89–1.52] in men and women respectively, interaction term p-value = 0.01).

**Conclusions:**

Rosiglitazone and pioglitazone appear to be associated with similar risks of SCA/VA.

## Background

Type 2 diabetes mellitus (DM) disproportionally affects persons of moderate to limited economic means [1]. Among the ~ 60% of the United States (US) population with a personal annual income < $48,000 [2], DM prevalence is 13–19%—2- to 3-fold greater than in persons with higher incomes [3]. From a global perspective, DM affects > 335 million residents of middle- to low-income countries [4] and its prevalence is increasing most rapidly in these nations [5]. Therefore, it is unsurprising that the World Health Organization deems cost a critical factor in type 2 DM treatment personalization [6]. In alignment with this, the American Diabetes Association and European Association for the Study of Diabetes has tailored guidelines [7] for cost-sensitive type 2 DM patients. Thiazolidinediones—generically available, low-cost insulin-sensitizing agents—are among the preferred add-ons to metformin for such patients without atherosclerotic cardiovascular or chronic kidney diseases. This highlights the continued role of thiazolidinediones in a practitioner’s toolkit of pharmacologic type 2 DM treatments.

Differential selectivity of thiazolidinediones for peroxisome proliferator-activated receptors in the myocardium may account for potential disparate effects on major cardiovascular events [8]. Numerous trials and meta-analyses have investigated relationships between thiazolidinediones and acute myocardial infarction (AMI) [9], stroke [9], dyslipidemia [10], left ventricular mass [11], heart failure [12], cardiovascular death [12], and all-cause death [13], as examples. To our knowledge, there have been no prior population-based studies of sudden cardiac arrest (SCA) and ventricular arrhythmia (VA). Such an investigation is warranted since high dose rosiglitazone may inhibit human ether-a-go-go-related gene (hERG) potassium channels (a surrogate for delayed cardiac repolarization [14]) in a human cell line [15]. Filling this knowledge gap is important because thiazolidinediones remain commonly used [16], may have synergistic effects with newer antidiabetic drugs [17], and may be repurposed for cancer [18, 19], neurodegenerative disorders [19], pulmonary arterial hypertension [20], fatty liver disease [19, 21], nephrotic syndrome [22], and secondary prevention of stroke [23]. Forthcoming trials examining thiazolidinediones and clinical sequelae of arrhythmogenicity are extremely unlikely given the futility of the TOSCA.IT trial [24] and the pharmacologic class’ lack of market exclusivity [16].

We therefore set forth to examine the relationship between individual thiazolidinediones and outpatient-originating SCA and VA.

## Methods

### Overview and study populations

We conducted high-dimensional propensity score (hdPS)-matched observational cohort studies to examine the risk of SCA/VA among new users of thiazolidinediones. The study included adults aged 30–75 years. Younger persons were excluded because SCA/VA is extremely rare in such individuals and unlikely to be due to prescription drugs [25]. Older persons were excluded to minimize concern for significant competing comorbidities that may mimic SCA/VA. The cohort consisted exclusively of person-time exposed to rosiglitazone or pioglitazone. Data included demographic, enrollment, and healthcare claims from the US Medicaid programs of California, Florida, New York, Ohio, and Pennsylvania from 1999 to 2012. These states comprise ~ 40% of the national Medicaid population, with the 14-year dataset recording the experience of nearly 70 million cumulative enrollees. Because many Medicaid beneficiaries are co-enrolled in the US Medicare program, we also obtained Medicare claims to ascertain a more complete picture of enrollees’ healthcare [26, 27]. We linked these datasets to the US Social Security Administration Death Master File to supplement dates of death with those provided by the US Centers for Medicare and Medicaid Services (CMS). For purposes of conceptual replication and robustness [28–30], and consistency with good practice for generating real-world evidence [31], we secondarily addressed this study question and examined the same estimands in an independent, 17-year US commercial health insurance dataset (Optum Clinformatics Data Mart, 2000–2016).

### Defining the cohort

Persons under study were apparent new users of a thiazolidinedione, i.e., had a 12-month baseline period devoid of a thiazolidinedione dispensing (including troglitazone [32] for 1999–2000). Cohort entry occurred upon an incident rosiglitazone or pioglitazone dispensing. The following 12-month baseline events served to exclude observations from study: (a) interruption in insurance benefit enrollment; and/or (b) SCA or VA diagnosis in an emergency department, inpatient, or ambulatory setting. We used the latter exclusion criterion to maximize the identification of incident outcomes described below. Persons with excluded observations could later be eligible for inclusion if subsequently meeting the above criteria; yet, once included, a person could not contribute second or later observations.

Follow-up began at cohort entry and continued until the first occurrence of a/an: (a) SCA or VA diagnosis, regardless of whether or not it met the outcome definition described below; (b) death (CMS only, since not recorded in Optum); (c) > 15-day gap in therapy for the cohort-defining thiazolidinedione; (d) dispensing of a thiazolidinedione different than that upon cohort entry (i.e., indicative of switching within pharmacologic class); (e) dispensing of a drug with a known risk of torsade de pointes; [33] (f) insurance benefit disenrollment; or (g) end date of the dataset. Although hospitalization was not a censoring event, we excluded follow-up time during a hospitalization to minimize immeasurable time bias [34].

### Exposure and covariate ascertainment

The thiazolidinedione dispensed on the day of cohort entry defined exposure. We did not study troglitazone given its US market withdrawal in 2000 [32]. To minimize the potential for selection bias and confounding by indication and other unmeasured subject characteristics [35], we did not study thiazolidinedione-unexposed persons. We selected pioglitazone as the active comparator referent since it: (a) is unlikely to prolong (although may shorten [36]) the electrocardiographic QT interval; [33] (b) is a predicted non-inhibitor of the human ether-a-go-go-related gene; [37] and (c) was utilized more frequently in these datasets, an important consideration for pairwise propensity score matching [38].

Potential confounders included prespecified and empirically identified baseline variables, both of which informed the propensity score. Prespecified variables included demographics, measures of intensity of healthcare utilization (e.g., numbers of prescription drugs used, healthcare provider visits, hospitalizations) [39], measures of socioeconomic status (Optum only), drug exposures, and comorbidities (Additional file 1: Table S1). Empiric variables were identified by a high-dimensional approach [40, 41] which ranks and selects potential confounders or proxies thereof based on their observed associations with exposure and outcome (see specifications in Additional file 1: Table S2).

### Outcome ascertainment

The outcome of primary interest was an incident *outpatient*-*originating* SCA/VA event precipitating hospital presentation—consistent with our aim to study the serious arrhythmogenic effects of thiazolidinediones in an ambulatory population. The rationale for a composite outcome is that SCA events are generally considered undocumented arrhythmias (i.e., sudden and presumed arrhythmic) [42]. We identified outcomes in emergency department or hospital claims having at least one discharge diagnosis code of interest (Additional file 1: Table S3) in the principal or first-listed position (indicative of the reason for presentation/admission) without regard to discharge disposition. The International Classification of Diseases, 9th Revision, Clinical Modification (ICD-9-CM) component of this algorithm was validated against primary medical records in a Medicaid population. These diagnoses had a positive predictive value (PPV) ~ 85% for identifying outpatient-originating SCA/VA [43]. The rationale for not using death certificate causes of death is that they have a poor PPV for identifying sudden death [44]. The rationale for not studying *inpatient*-*originating* SCA/VA is that: (a) oral antidiabetic drugs are rarely utilized in the inpatient setting; [45] (b) arrhythmogenic events occurring during hospitalizations are often attributable to causes other than ambulatory drug exposures; and c) CMS and Optum data, like most claims datasets, do not record inpatient drug exposures [46].

The outcome of secondary interest was the subset of primary events that were fatal, i.e., sudden cardiac death (SCD) or fatal VA. Operationally, this was defined as having died the day of or the day after the healthcare encounter defining the event.

### Statistical analysis

We calculated descriptive statistics for baseline variables, crude incidence rates, and unadjusted association measures, the latter via Cox proportional hazards models. We utilized a semi-automated, data-adaptive hdPS approach—an algorithm for identifying and selecting proxies for important confounder constructs [47]—to reduce the impact of measured and unmeasured potential confounders that are correlated with measured factors [48]. First, we used the hdPS algorithm [41, 47] to identify empiric candidate covariates; we identified the 200 most prevalent baseline diagnoses, procedures, and dispensed drugs for each of nine prespecified data dimensions. Second, within each dimension, we ranked candidates based on their potential for bias by assessing each variable’s prevalence and univariate association with exposure and outcome according to the Bross formula [49, 50]. Third, we used these associations to select 500 empiric covariates for inclusion in the propensity score. We also included in the propensity score: demographics; measures of intensity of healthcare utilization; [51] and investigator-prespecified covariates meeting the disjunctive cause criterion (Additional file 1: Table S1) [52]. We assessed covariate balance between thiazolidinedione groups using standardized differences [53]. Fourth, we used logistic regression to calculate propensity scores, defined as a subject’s predicted probability of receiving rosiglitazone vs. pioglitazone. Fifth, we paired rosiglitazone to pioglitazone users (1:1) on propensity score using nearest-neighbor caliper (width = 0.2 standard deviations of the logit of the propensity score) matching without replacement; matching began with study subjects in a random order [54]. Sixth, we generated Kaplan–Meier curves and compared their equality using a stratified log-rank test [38]. Finally, we generated marginal hazard ratios (HRs) via Cox proportional hazards regression that adjusted for calendar time and used a robust variance estimator to account for clustering within matched pairs [38, 55]. We assessed proportional hazards assumptions via inclusion of an interaction term of exposure by time.

We conducted numerous secondary analyses (e.g., dose–response) to assess the robustness of our primary findings (Additional file 1: Table S4). Primary and secondary analyses were conducted using SAS v9.4 (SAS Institute Inc.: Cary, NC). The University of Pennsylvania institutional review board approved this research.

### Role of the funding sources

Neither the American Diabetes Association nor the US National Institutes of Health had a role in the study’s conduct or interpretation.

## Results

### Cohort characteristics and outcome frequencies|Medicaid

In the Medicaid dataset, we identified 294,324 and 205,767 new users of pioglitazone and rosiglitazone, respectively. Additional file 1: Table S5 and Figure S1 display their baseline characteristics. Overall, users were predominantly female (62.2%) and non-Hispanic white (34.9%), with a median age of 58.7 years. Large proportions of users had pre-existing hypertension (65.2%), dyslipidemia (50.2%), depression (26.1%), and ischemic heart disease (23.4%). Small proportions of users had pre-existing cardiomegaly (5.7%), a cardiac conduction disorder (1.9%), and a congenital cardiac anomaly (1.6%). Few users experienced a prior serious hypoglycemic episode (2.8%).

Users contributed 184,664 person-years (p-y) of follow-up, during which we identified 528 SCA/VA outcomes (crude incidence rate = 2.86 [95% confidence interval: 2.62–3.11] per 1000 p-y), 273 (51.7%) of which were fatal. See Additional file 1: Figure S2 for the Kaplan–Meier curve. In the secondary analysis limited to the first 30 days of follow-up, we identified 215 SCA/VA outcomes during 35,592 p-y of follow-up (crude incidence rate = 6.04 [5.26–6.90] per 1000 p-y). Crude incidence rates for SCD/fatal VA were 1.48 (1.31–1.66) and 3.26 (2.69–3.91) per 1000 p-y in all follow-up time and limited to the first 30 days of follow-up, respectively. These incidence rates are similar to prior findings in persons with DM [56–58].

### Effect estimates: primary analysis|Medicaid

**Fig. 1:**
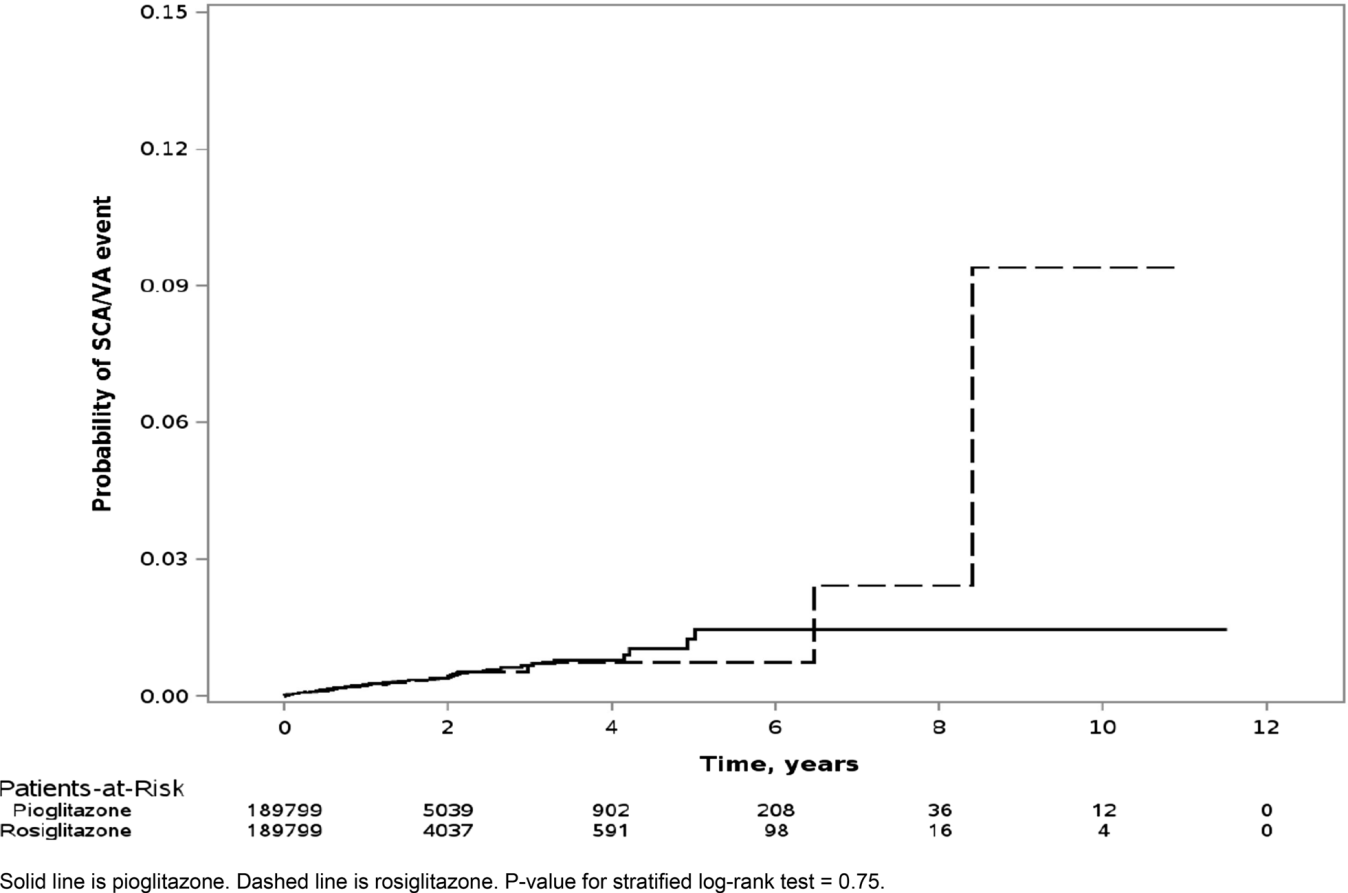
Kaplan–Meier curve depicting the probability of sudden cardiac arrest/ventricular arrhythmia upon new use of rosiglitazone vs. pioglitazone, limited to the propensity score-matched sample in Medicaid (N = 379,598). Solid line is pioglitazone. Dashed line is rosiglitazone. p-value for stratified log-rank test = 0.75

**Fig. 2:**
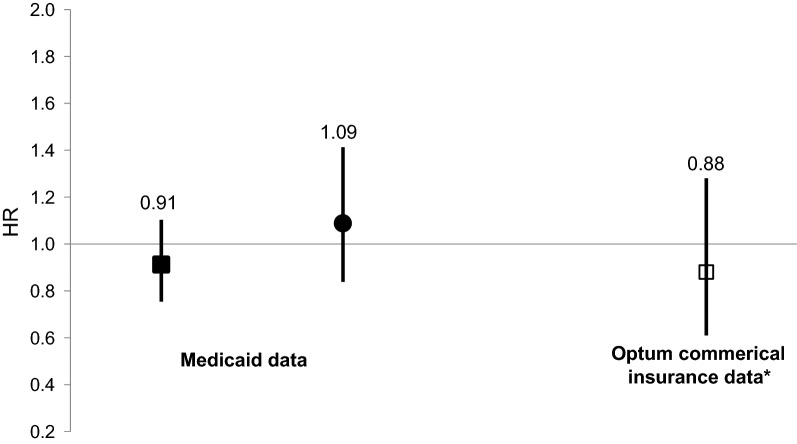
Confounder-adjusted marginal hazard ratios for rosiglitazone (vs. pioglitazone) exposure and primary and secondary outcomes, by dataset | Medicaid and Optum. *HR* hazard ratio. Squares depict hazard ratios for the primary outcome of sudden cardiac arrest and ventricular arrhythmia. The circle depicts a hazard ratio for the secondary outcome of sudden cardiac death and fatal ventricular arrhythmia. * Optum was the prespecified conceptual replication dataset. Its analyses were limited to the primary outcome since the dataset does not document deaths

The propensity score model included 560 covariates—60 prespecified and 500 empirically identified by the hdPS algorithm (Additional file 1: Table S6). Using logits of propensity scores, we matched 189,799 rosiglitazone users (92.2% of available population) to an equal number of pioglitazone users (N_Total_ = 379,598). Additional file 1: Figure S3 depicts near-perfect overlap in propensity score distributions post-matching. Figure 1 presents the Kaplan–Meier curve in the matched sample. Table 1 presents unadjusted HRs. Table 1 and Fig. 2 present adjusted marginal HRs. Notably, the hazard of SCA/VA for rosiglitazone (vs. pioglitazone) was consistent with the null (adjusted marginal HR = 0.91 [0.75–1.10]).

**Table 1:** Outcomes and effect estimates for the primary analysis|Medicaid

Outcomes during follow-up period	Thiazolidinedione	
	Pioglitazone	Rosiglitazone
	N (%)	
Sudden cardiac arrest/ventricular arrhythmia	295	233
Sudden cardiac arrest	217 (73.6)	175 (75.1)
Ventricular arrhythmia	60 (20.3)	37 (15.9)
Both (contemporaneously)	18 (6.1)	21 (9.0)
Sudden cardiac arrest/ventricular arrhythmia immediately preceded^a^ by hospitalization for an acute ischemic event	0 (0.0)	^c^
Sudden cardiac arrest/ventricular arrhythmia immediately preceded^a^ by emergency department presentation or hospitalization for hypoglycemia	^c^	^c^

Measure of sudden cardiac arrest/ventricular arrhythmia occurrence	Incidence rate (95% confidence interval)	
Unadjusted, per 1000 person-years	2.67 (2.37–2.99)	3.14 (2.75–3.57)
Age- and sex-standardized^b^, per 1000 person-years	2.89 (2.55–3.24)	3.38 (2.92–3.85)

Relative effect estimates for sudden cardiac arrest/ventricular arrhythmia	Hazard ratio (95% confidence interval)	
Unadjusted^†^	1.00 (referent)	1.16 (0.98–1.38)
Confounder-adjusted^‡^, also see ■ in Fig. 2	1.00 (referent)	0.91 (0.75–1.10)

^a^Operationalized as an event within the 3 days preceding hospital presentation for sudden cardiac arrest/ventricular arrhythmia

^b^Direct standardization using age-by-sex distribution of thiazolidinedione users identified in 2005–2012 National Ambulatory Medical Care Survey (Centers for Disease Control and Prevention: Atlanta, Georgia)

^c^Omitted in compliance with Centers for Medicare and Medicaid Services data privacy policy (i.e., prohibition of reporting cell counts < 11)

^†^Did not fail a test for non-proportional hazards, p = 0.62

^‡^Did not fail a test for non-proportional hazards, p = 0.92

### Effect estimates: secondary analyses|Medicaid

Effect estimates from secondary analyses of the Medicaid dataset (Table 2) were consistent with the primary finding. We found treatment effect heterogeneity by sex (adjusted marginal HR among men = 0.71 [0.54–0.93], among women = 1.16 [0.89–1.52], interaction term p-value = 0.01). Other secondary analyses examining high-risk subgroups did not meet the prespecified threshold for statistical significance. Further, we did not identify dose–response relationships (Additional file 1: Figure S4).

**Table 2:** Summary of results from secondary analyses | Medicaid and Optum

Analysis,^a^ prespecified and conducted in Medicaid unless otherwise noted	Results		
	N	aHR for rosiglitazone^b^ and sudden cardiac arrest/ventricular arrhythmia	aHR for rosiglitazone^b^ and sudden cardiac death/fatal ventricular arrhythmia
Limiting maximum follow-up time to 30 days	379,598	0.91 (0.67–1.23)	1.09 (0.73–1.64)
Limiting maximum follow-up time to 6 years (post hoc)	379,598	0.90 (0.74–1.09)	1.08 (0.83–1.40)
Limiting study period to time before January 1, 2007 (post hoc)	315,196	0.90 (0.74–1.11)	1.00 (0.76–1.30)
Decreasing permissible grace period between contiguous thiazolidinedione dispensings from 15 to 7 days	379,598	0.95 (0.77–1.18)	1.17 (0.88–1.56)
Increasing permissible grace period between contiguous thiazolidinedione dispensings from 15 to 30 days	379,598	0.97 (0.81–1.15)	1.08 (0.83–1.40)
Excluding, as a censoring criterion, the occurrence of a VA diagnosis not meeting the outcome definition	379,598	0.91 (0.75–1.10)	1.08 (0.84–1.41)
Exclusion of persons with an any-claim type, any-position diagnosis of SCA or VA ever prior to cohort entry	374,694	0.89 (0.73–1.08)	1.05 (0.80–1.37)
Exclusion of empiric covariates from the PS thought to be strong correlates of exposure but not associated with the outcome	384,976	0.90 (0.75–1.10)	1.08 (0.83–1.41)
Limiting outcomes to fatal events	379,598	–	1.09 (0.84–1.41)
Examining thiazolidinedione dose–response relationships and limiting maximum follow-up time to 90 days	See Additional file 1: Figure S4		
Examining the same estimands in an independent, commercial health insurance dataset (Optum Clinformatics Data Mart, 2000–2016), also see □ in Fig. 2	195,742	0.88 (0.61–1.28)	Not applicable, as our Optum dataset does not record death in any setting

Examining effect modification by	N	p-value for interaction term	aHR for rosiglitazone† and sudden cardiac arrest/ventricular arrhythmia
Concomitant use of drugs that inhibit hepatic CYP450-based metabolism of thiazolidinediones			
CYP2C8 inhibitors	379,598	0.10	Since the interaction term p-values did not meet the prespecified threshold for statistical significance, stratified results are not presented
CYP2C9 inhibitors	379,598	0.95	
CYP3A4 inhibitors	379,598	0.69	
Concomitant use of drugs with a “known risk of TdP”	379,598	0.44	
Concomitant use of drugs with a “known”, “possible”, or “conditional risk of TdP”	379,598	0.18	
Other high-risk subgroups			
Age group	379,598	0.86	
Sex	379,598	0.01	Among women: 1.16 (0.89–1.52) Among men: 0.71 (0.54–0.93)
Race	379,598	0.56	Since the interaction term p-values did not meet the prespecified threshold for statistical significance, stratified results are not presented
Nursing home residence	379,598	0.72	
Ischemic heart disease	379,598	0.58	
Conduction disorders	379,598	0.36	
HF/cardiomyopathy	379,598	0.92	
Kidney disease	379,598	0.38	
Liver disease	379,598	0.86	

*aHR* adjusted hazard ratio, *CYP* cytochrome P450, *HF* heart failure, *N* number of thiazolidinedione users under study, *PS* propensity score, *SCA* sudden cardiac arrest, *SCD* sudden cardiac death, *TdP* torsade de pointes, *VA* ventricular arrhythmia

^a^Rationales for these secondary analyses are detailed in Additional file 1: Table S4

^b^Versus pioglitazone as prespecified referent

### Conceptual replication [28–30]|Optum

In the Optum dataset, we identified 190,226 and 103,834 new users of pioglitazone and rosiglitazone, respectively. In contrast to Medicaid, users in Optum were predominantly male (56.5%), had a higher burden of dyslipidemia (60.6%), and had lower burdens of depression (13.8%), ischemic heart disease (13.3%), and prior serious hypoglycemia (0.6%). The crude incidence rate of SCA/VA was 1.41 (1.21–1.64) per 1000 p-y, approximately half the rate estimated among Medicaid enrollees. Consistent with our Medicaid finding, the hazard of SCA/VA for rosiglitazone (vs. pioglitazone) was consistent with the null (adjusted marginal HR = 0.88 [0.61–1.28]). Unlike in Medicaid, we did not find treatment effect heterogeneity by sex (interaction term p-value = 0.96). Further, we did not identify dose–response relationships (Additional file 1: Figure S5).

## Discussion

This post-market comparative safety study using real-world healthcare data is the first to estimate effects of thiazolidinediones on SCA/VA as a stand-alone endpoint. The crude incidence rate of SCA/VA among thiazolidinedione users (2.86 per 1000 p-y) reported herein is less than we previously found among sulfonylurea users (3.57 per 1000 p-y); [58] this may be partly driven by different rates of serious hypoglycemia between these pharmacologic classes [59, 60]. Our overall finding of no difference in SCA/VA between new users of rosiglitazone vs. pioglitazone (adjusted marginal HR = 0.91) was robust across numerous secondary analyses and conceptually replicated in an independent dataset (adjusted marginal HR = 0.88). This null finding aligns with a human cell biology study demonstrating that rosiglitazone’s inhibition of hERG (a putative SCA surrogate [14]) is limited to supratherapeutic doses (half maximal inhibitory concentration ~ 9- to 19-fold therapeutic human plasma concentration) [15]. Interestingly, our examination of potential effect modifiers found potential SCA differences by sex among Medicaid, but not Optum, enrollees. The Medicaid finding is consistent with prior reports of sex-based heterogeneity in thiazolidinedione effects, including on hemoglobin A1c, weight gain, edema, and AMI [61, 62].

SCA is a common and growing problem in type 2 DM given the interrelatedness of abnormalities in glucose/insulin homeostasis, dyslipidemia, coronary atherosclerosis, myocardial fibrosis, and QT interval prolongation [57]. In this population, 70% of deaths are attributed to cardiovascular disease, half of which are SCAs [63]. Saxagliptin assessment of vascular outcomes recorded in patients with diabetes mellitus–thrombolysis in myocardial infarction 53 (SAVOR–TIMI 53) data suggest that hemoglobin A1c is a unique predictor of sudden cardiac, but not other, deaths [64]. Therefore, insulin-sensitizing, glucose-lowering, and/or pleiotropic effects of antidiabetic medications may reduce risks of cardiovascular morbidity and mortality. Despite early reports of beneficial effects on atherosclerotic processes, cardiovascular sequelae of thiazolidinediones remain incompletely understood [11, 24, 65, 66]. Despite rosiglitazone’s fall from favor [67], our examination remains clinically relevant given (a) reassuring findings on cardiovascular death, AMI, and stroke endpoints from a re-analysis of RECORD; [68] and (b) its lack of an association with bladder cancer [69].

Among all potential cardiovascular effects, we investigated SCA/VA given the paucity of data on the topic. The recently terminated TOSCA.IT pragmatic trial attempted to compare sudden death in concomitant users of metformin and pioglitazone, but was underpowered [24]. A cohort study using i3 (now Optum) data examined sudden death as part of a composite secondary endpoint with rosiglitazone vs. pioglitazone, but did not report sudden death specific findings [70]. The following biologic underpinnings supported our decision to elucidate *within*-*pharmacologic class* SCA/VA risks and thereby created clinical equipoise. First, a molecular biology study found that high-dose rosiglitazone but not pioglitazone [36] inhibited hERG [15]. Second, rosiglitazone lacks pioglitazone’s favorable actions on lipids, serum measures of which are evident within the first 4 weeks of therapy [71]. Despite these apparent distinctions, we found no overall difference in SCA/VA risk between new users of rosiglitazone and pioglitazone.

Motivated by prior findings that sex may alter risk–benefit considerations among thiazolidinedione users [62], we prespecified, examined, then observed effect modification by sex in Medicaid enrollees. Among men, SCA/VA risk was 29% lower among new users of rosiglitazone vs. pioglitazone; the finding among women was consistent with a null association. The potential differential risk by sex, if real, may be at least partly explained by differential responses to thiazolidinediones (e.g., via hormonal mechanisms, peroxisome proliferator-activated receptor expression) [72], hypoglycemia rates [73], and cardiovascular effects [74], as examples. The apparent protective association for rosiglitazone in men may be explained by its less potent effect on plasma glucose [75], subsequent titration to higher doses, and lower rates of serious hypoglycemia at these doses [59], all vs. pioglitazone, for example. Further investigation of this result is prudent, especially since our sex finding did not replicate in Optum enrollees.

Our study has notable strengths. It is the first population-based study to examine the relationship between thiazolidinediones and SCA/VA. Such results are not forthcoming from ongoing trials and, given the futility of TOSCA.IT, are unlikely to be examined in future trials. Our algorithm to identify the clinical outcome of interest was developed and validated in a population used herein and has a good PPV [43]. Our implementation of an incident user design, active comparator reference exposure, data adaptive approach to identify then adjust for confounders and their proxies, and secondary analyses served to mitigate confounding. Finally, we estimated marginal treatment effects, the same type of measure that arises from a clinical trial.

Our study also has limitations. First, despite rigor in our design and analysis, residual differences between pioglitazone and rosiglitazone users may remain. Second, our lack of access to biosamples precluded an examination of genetic determinants of SCA/VA risk. Third, our adjustment for family history of diseases relied on diagnostic coding and therefore was likely underascertained. Fourth, we lacked data on direct adherence to thiazolidinedione therapy. To address this, we conducted secondary analyses in which we modified the allowable grace period between contiguous prescriptions. Fifth, we did not assess competing events that may have precluded our observation of the outcome [76]. If competing risks were present, our reliance on the Kaplan–Meier estimate of the survival function to estimate the incidence function would generally result in an upward bias in the estimation of the incidence function [77]. Relatedly, we did not model cause-specific hazard functions. Finally, we may have underascertained outcomes, likely biasing towards the null. Because SCA/VA was defined using emergency department and inpatient diagnosis codes, we likely missed fatal events not resulting in hospital presentation. However, prior work suggests that 69–80% of persons experiencing an out-of-hospital cardiac arrest [78, 79] and up to 88% of persons experiencing a witnessed ventricular tachycardia survive to hospital admission [80], although recent registry data from CARES suggests poorer survival-to-admission rates (18–49%, depending on presenting characteristics) [81]. We considered using death certificates to identify supplemental events, but decided against it given the approach’s poor PPV for identifying SCA/VA [44, 82, 83].

## Conclusions

Thiazolidinediones are a low-cost, effective treatment for type 2 DM, a highly prevalent condition in persons with limited economic means. Although substantial attention has been paid to thiazolidinediones and risk of AMI and heart failure, there is a major knowledge gap in their arrhythmogenic safety. In response, we generated real-world evidence that rosiglitazone and pioglitazone have similar risks of SCA/VA.

## Supplementary information

**Additional file 1.** Additional tables and figures.

## Data Availability

The data that support the findings of this study are available from the United States (US) Centers for Medicare and Medicaid Services (CMS) and Optum Inc., but restrictions apply to the availability of these data, which were used under license for the current study and so are not publicly available. Data may be available from the authors upon reasonable request and with explicit permission from CMS and Optum Inc.

## Abbreviations

AMI: Acute myocardial infarction
CARES: Cardiac arrest registry to enhance survival
CMS: Centers for Medicare and Medicaid Services
DM: Diabetes mellitus
hdPS: High-dimensional propensity score
hERG: Human ether-a-go-go-related gene
HR: Hazard ratio
ICD-9-CM: International classification of diseases, 9th revision, clinical modification
PPV: Positive predictive value
P-Y: Person-years
RECORD: Rosiglitazone evaluated for cardiovascular outcomes in oral agent combination therapy for type 2 diabetes
SAVOR–TIMI 53: Saxagliptin assessment of vascular outcomes recorded in patients with diabetes mellitus–thrombolysis in myocardial infarction 53
SCA: Sudden cardiac arrest
SCD: Sudden cardiac death
TOSCA.IT: Thiazolidinediones or sulfonylureas and cardiovascular accidents intervention trial
US: Unites States
VA: Ventricular arrhythmia
