# Supplementary material for: The risk of sudden cardiac arrest and ventricular arrhythmia with rosiglitazone versus pioglitazone: real-world evidence on thiazolidinedione safety
Source: Cardiovasc Diabetol. 2020 Feb 25;19:25. doi: 10.1186/s12933-020-00999-5 (PMC7041286; doi:10.1186/s12933-020-00999-5)
Supplement: Supplementary file 1 — Additional file 1. Additional tables and figures. [file 12933_2020_999_MOESM1_ESM.docx]

### Additional file 1

### Additional file 1. Table S1. Prespecified covariates included in the propensity score

| Demographics | age | |
| --- | --- | --- |
|  | sex | |
|  | race | |
|  | state of residence | |
|  | enrollment in Medicare | |
|  | enrollment in Medicaid | |
|  | residence in long-term care | |
| Socioeconomic status* | education level | |
|  | federal poverty status | |
|  | home ownership | |
|  | household income | |
| Measures of intensity of healthcare utilization | numbers of: prescriptions dispensed; inpatient diagnoses; inpatient procedures; outpatient diagnoses; outpatient procedures; other setting diagnoses; other setting procedures | |
|  | polypharmacy, defined as ≥5 drug dispensings for unique active ingredients | |
| Comorbidities | alcohol abuse | |
|  | cardiomegaly | |
|  | conduction disorders | |
|  | congenital anomalies of the heart | |
|  | depression | |
|  | diabetes complications severity index, adapted^1^, as continuous measure^2^ | |
|  | heart disease, chronic rheumatic | |
|  | heart disease, ischemic | |
|  | heart failure / cardiomyopathy | |
|  | hypertensive disease | |
|  | hypoglycemia, serious, i.e., precipitating emergency department and/or inpatient care | |
|  | implantable cardioverter defibrillator/pacemaker use | |
|  | kidney disease | |
|  | lipid metabolism disorders | |
|  | liver disease | |
|  | obesity | |
|  | osteoporosis | |
|  | sudden cardiac death, family history* | |
|  | tobacco use | |
| Concomitant drug use | antidiabetic drugs | alpha glucosidase inhibitors |
|  |  | amylin analogs |
|  |  | dipeptidyl peptidase-4 inhibitors |
|  |  | glucagonlike peptide 1 receptor agonists |
|  |  | insulins |
|  |  | metformin |
|  |  | meglitinides |
|  |  | sulfonylureas |
|  | drugs with clinically relevant^3^ impact on CYP isozymes | CYP2C9 inhibitors |
|  |  | CYP3A4 inhibitors |
|  |  | CYP2C8 inhibitors |
|  |  | CYP2C9 inducers |
|  |  | CYP3A4 inducers |
|  | drugs with TdP risk^4^ | drugs with “known” risk of TdP |
|  |  | drugs with “known,” “possible,” or “conditional risk of TdP |
| Laboratory measures* | creatinine, serum** | |
|  | glucose, blood*** | |
|  | hematocrit | |
|  | hemoglobin | |
|  | hemoglobin A1c | |
|  | hypoglycemia, alert value,^5^ defined by blood glucose ≤10-70mg/dL | |
|  | hypoglycemia, clinically significant,^5^ defined by blood glucose <10-54mg/dL | |
| CYP = cytochrome P450;dL = deciliter; mg = milligrams; TdP = torsade de pointes  * Optum analyses only  ** Glomerular filtration rate measures were empirically identified by high-dimensional approach and included in the propensity score (Optum analyses only)  *** Included fasting and nonfasting measurements  1. Glasheen et al. Diabetes Complications Severity Index (DCSI)-Update and ICD-10 translation. *J Diabetes Complications* 2017;31(6):1007-1013.  2. Young et al. Diabetes complications severity index and risk of mortality, hospitalization, and healthcare utilization. *Am J Manag Care* 2008;14(1):15-23.  3. Flockhart et al. The Flockhart Table™. Drug Interactions: Cytochrome P450 drug interaction table. Indiana University School of Medicine. 2019.  4. Woosley et al. CredibleMeds.org: What does it offer? *Trends Cardiovasc Med* 2018;28(2):94-99.  5. International Hypoglycaemia Study Group. Glucose concentrations of less than 3.0 mmol/L (54 mg/dL) should be reported in clinical trials: A joint position statement of the American Diabetes Association and the European Association for the Study of Diabetes. *Diabetes Care* 2017;40(1):155-157. | | |

### Additional file 1. Table S2. Specifications used in the empiric identification of covariates for propensity score inclusion

| Data dimensions (*p*) | Inpatient^1^ ICD-CM diagnoses |
| --- | --- |
|  | Inpatient^1^ ICD-CM/PCS procedures |
|  | Inpatient^1^ CPT/HCPCS procedures |
|  | Laboratory^2^ LOINC observations |
|  | Outpatient^3^ ICD-CM diagnoses |
|  | Outpatient^3^ ICD-CM/PCS procedures |
|  | Outpatient^3^ CPT/HCPCS procedures |
|  | Other setting^4^ ICD-CM diagnoses |
|  | Other setting^4^ ICD-CM/PCS procedures |
|  | Outpatient* medication^5^ active ingredients |
| Granularity of *p* | 3 digits for ICD-9-CM diagnoses, 3 characters for ICD-10-CM diagnoses, 7 characters for LOINC observations, 2 digits for ICD-9-CM procedures, 3 characters for ICD-10-PCS procedures, 5 digits for CPT, 5 characters for HCPCS, and Cerner Multum (Cerner Corporation: Kansas City, MO) Lexicon Plus-defined active ingredient for drugs |
| Empiric covariates identified (*n*), per *p*, ranked in descending order by prevalence | *n* = 200 |
| Method of covariate prioritization | Bross bias formula:  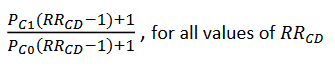  where P_C1_ represents the prevalence of the binary covariate within the group with the exposure of interest, P_C0_ the prevalence of the binary covariate within the group with the reference exposure, and RR_CD_ the relative risk for the univariate association between the binary covariate and the outcome.^6^ |
| Empiric covariates selected (*k*),across *p*, subsequent to prioritization | *k* = 500, plus the investigator-predefined covariates listed in **Additional file 1.** **Table S1** |
| Zero-cell correction screening | No, given adequate number of exposed outcomes |
| CM = Clinical Modification; CPT = Current Procedural Terminology; HCPCS = Healthcare Common Procedure Coding System; ICD = International Classification of Diseases; LOINC = Logical Observation Identifiers Names and Codes; PCS = Procedure Coding System  1. For Centers for Medicare and Medicaid Services: claims arising from Medicaid Analytic Extract Inpatient file and Medicare Provider Analysis and Review file (limited to short stay and long stay hospitalization claims). For Optum: claims arising from Medical or Confinement tables with place of service = inpatient hospital.  2. Not applicable to Centers for Medicare and Medicaid Services. For Optum: claims arising from Laboratory table.  3. For Centers for Medicare and Medicaid Services: claims arising from Medicaid Analytic Extract Other Therapy file, Medicare Carrier file, and Medicare Outpatient Standard Analytic File. For Optum: claims arising from Medical or Confinement tables with place of service other than inpatient hospital or facility listed in footnote 4.  4. For Centers for Medicare and Medicaid Services: claims arising from Medicaid Analytic Extract Long Term Care file and Medicare Provider Analysis and Review file (limited to skilled nursing facility claims). For Optum: claims arising from Medical or Confinement tables with place of service = assisted living facility, group home, skilled nursing facility, nursing facility, custodial care facility, inpatient psychiatric facility, intermediate care facility, residential substance facility, psychiatric residential treatment facility, comprehensive inpatient rehabilitation facility, or comprehensive outpatient rehabilitation facility.  5. For Centers for Medicare and Medicaid Services: claims arising from Medicaid Analytic Extract Prescription file and Medicare Part D Event Data file. For Optum: claims arising from Prescription table.  6. Wyss et al. Erratum: High-dimensional propensity score adjustment in studies of treatment effects using health care claims data. *Epidemiology*. 2018;29(6):e63-e64.  * Inpatient medications are not reported in Centers for Medicare and Medicaid Services or Optum data | |

**Additional file 1.** **Table S3. Operational definition for the composite outcome of interest**

| **Outcome component** | **Diagnosis** | **ICD-9-CM discharge diagnosis code*** | **ICD-10-CM discharge diagnosis code**** | **Discharge diagnosis position and claim type** |
| --- | --- | --- | --- | --- |
| sudden cardiac arrest | cardiac arrest | 427.5 | I46.2  I46.8  I46.9 | First-listed discharge diagnosis on an emergency department claim or a principal discharge diagnosis on an inpatient hospitalization claim |
|  | sudden death, cause unknown | 798 | - |  |
|  | instantaneous death | 798.1 | - |  |
|  | death occurring in less than 24 hours from onset of symptoms, not otherwise explained | 798.2 | - |  |
| ventricular arrhythmia | (paroxysmal) ventricular tachycardia | 427.1 | I47.2 |  |
|  | ventricular fibrillation and flutter | 427.4 | I49.0 |  |
|  | ventricular fibrillation | 427.41 | I49.01 |  |
|  | ventricular flutter | 427.42 | I49.02 |  |
| CM = Clinical Modification; ICD = International Classification of Diseases  *ICD-9-CM codes used for January 1, 1999 through December 31, 2012 in Centers for Medicare and Medicaid Services data and January 1, 2000 through September 30, 2005 in Optum data  **ICD-10-CM codes used for October 1, 2015 through December 31, 2016 in Optum data; the conversion to ICD-10-CM from ICD-9-CM was achieved via forward-backward general equivalence mapping^1^ and subsequently vetted by a cardiac electrophysiologist (R.D.)  1. Fung et al. Preparing for the ICD-10-CM transition: Automated methods for translating ICD codes in clinical phenotype definitions. *EGEMS (Wash DC)* 2016;4(1):1211. | | | | |

**Additional file 1. Table S4. Secondary analyses**

| **Analysis*** | **Rationale** |
| --- | --- |
| ***Minimizing the role of chance, bias, and/or confounding*** | |
| Limiting maximum follow-up time to 30 days | Restricts follow-up period to that immediately after the incident thiazolidinedione exposure, thereby minimizing depletion of susceptible person-time and concern for informative censoring. |
| Limiting maximum follow-up time to 6 years (*post hoc*) | Restricts follow-up period to time before crossing of Kaplan-Meier survival curves |
| Limiting study period to time before January 1, 2007 (*post hoc*) | Restricts study period to calendar time predating major thiazolidinedione warnings,^1^ thereby minimizing concern for channeling. |
| Decreasing permissible grace period** between contiguous thiazolidinedione dispensings from 15 days to 7 days | Reduces proportion of follow-up time contributed by periods of presumed nonadherence to thiazolidinedione therapy, thereby minimizing potential exposure misclassification |
| Increasing permissible grace period** between contiguous thiazolidinedione dispensings from 15 days to 30 days | Increases proportion of follow-up time contributed by periods of presumed nonadherence to thiazolidinedione therapy, thereby minimizing premature censoring of follow-up among poor adherers |
| Excluding, as a censoring criterion, the occurrence of a VA diagnosis not meeting the outcome definition | Minimizes concern for censoring observation time by a condition that may lie along the causal pathway between exposure and outcome |
| Exclusion of persons with an any-claim type, any-position diagnosis of SCA or VA ever prior to cohort entry | Expands exclusion of persons with a prior history of SCA/VA from 12 months to ever prior to cohort entry, thereby ensuring the study of incident events in persons with no prior history of the outcome |
| Exclusion of empiric covariates from the PS thought to be strong correlates of exposure but not associated with the outcome | Restricts PS covariates to non-instruments, as inclusion of an instrumental variable in a PS can increase standard error and bias, thereby minimizing standard error and bias |
| ***Further elucidating the association between exposure and outcome*** | |
| Limiting maximum follow-up time to 30 days | Facilitates elucidation of an immediate effect during a presumptive high-risk period |
| Limiting outcomes to fatal events | Facilitates elucidation of risks of the most serious outcomes |
| Examining thiazolidinedione dose-response relationships and limiting maximum follow-up time to 90 days^†^ | Facilitates examining initial thiazolidinedione dose during the period in which post-cohort entry dose titration has yet to occur |
| Examining effect modification by drugs that inhibit hepatic CYP450-based metabolism of thiazolidinediones^2-6^ | Functionally, an alternative method of examining dose-response; facilitates examination of treatment effect heterogeneity |
| Examining effect modification by drugs that have a “known risk of TdP”^6^ | Facilitates examination of treatment effect heterogeneity |
| Examining effect modification by drugs that have a “known”, “possible”, or “conditional risk of TdP”^6^ | Facilitates examination of treatment effect heterogeneity |
| Examining effect modification by other high-risk subgroups^††^ | Facilitates examination of treatment effect heterogeneity |
| ***For purposes of conceptual replication and robustness^8-10^*** | |
| Examining the same estimands in an independent, commercial health insurance dataset (Optum Clinformatics Data Mart, 2000–2016) | Allows for assessment of ability to obtain similar findings with application of the same design and methodologic choices in a different data source |
| CYP = cytochrome P450; PS = propensity score; SCA = sudden cardiac arrest; TdP = torsade de pointes; VA = ventricular arrhythmia | |
| * prespecified unless otherwise noted  ** days added to the end of a prescription claim’s days’ supply field to account for nonadherence to prescribed therapy | |
| † dose calculated as milligram-quantity of the prescription divided by the days’ supply | |
| †† age strata,^11^ sex, race, nursing home residence, ischemic heart disease, conduction disorders, heart failure / cardiomyopathy, kidney disease, and liver disease | |
| 1. Starner et al. Rosiglitazone and pioglitazone utilization from January 2007 through May 2008 associated with five risk-warning events. *JMCP* 2008;14(6):523-531.  2. Baldwin et al. Characterization of the cytochrome P450 enzymes involved in the in vitro metabolism of rosiglitazone. *Br J Clin Pharmacol* 1999;48(3):424-432. | |
| 3. Jaakkola et al. Pioglitazone is metabolised by CYP2C8 and CYP3A4 in vitro: Potential for interactions with CYP2C8 inhibitors. *Basic Clin Pharmacol Toxicol* 2006;99(1):44-51. | |
| 4. Kajosaari et al. Pioglitazone, an in vitro inhibitor of CYP2C8 and CYP3A4, does not increase the plasma concentrations of the CYP2C8 and CYP3A4 substrate repaglinide. *Eur J Clin Pharmacol* 2006;62(3):217-223. | |
| 5. Kirchheiner et al. Effect of genetic polymorphisms in cytochrome p450 (CYP) 2C9 and CYP2C8 on the pharmacokinetics of oral antidiabetic drugs: Clinical relevance. *Clin Pharmacokinet* 2005;44(12):1209-1225. | |
| 6. Sahi et al. Comparative effects of thiazolidinediones on in vitro P450 enzyme induction and inhibition. *Drug Metab Disposition* 2003;31(4):439-446. | |
| 7. Woosley et al. CredibleMeds.org: What does it offer? *Trends Cardiovasc Med* 2018;28(2):94-99.  8. Wang et al. Reporting to improve reproducibility and facilitate validity assessment for healthcare database studies V1.0. *Pharmacoepidemiol Drug Saf* 2017;26(9):1018-1032.  9. Wang et al. Reporting to improve reproducibility and facilitate validity assessment for healthcare database studies V1.0. *Value Health* 2017;20(8):1009-1022.  10. Wang et al. Transparency and reproducibility of observational cohort studies using large healthcare databases. *Clin Pharmacol Ther* 2016;99(3):325-332.  11. Becker et al. Racial differences in the incidence of cardiac arrest and subsequent survival. The CPR Chicago Project. *N Engl J Med* 1993;329:600-6. | |

**Additional file 1.** **Table S5. Characteristics of thiazolidinedione users | Medicaid**

|  | | | **Unmatched cohort** | | | **PS matched cohort** | | |
| --- | --- | --- | --- | --- | --- | --- | --- | --- |
|  |  |  | **pioglitazone** | **rosiglitazone** | | **pioglitazone** | **rosiglitazone** | |
|  | | | N (unless otherwise noted) | | | | | |
| Users | | | 294,324 | 205,767 | | 189,799 | 189,799 | |
| Person-years of follow-up, sum, among all users | | | 110,527 | 74,138 | | 72,793 | 68,268 | |
| Days of follow-up, median (5th, 95th percentile), per user | | | 70 (1, 508) | 69 (1, 477) | | 70 (1, 523) | 68 (1, 476) | |
| PDCs (an adherence measure) for thiazolidinedione dispensings, median (5th, 95th percentile) | | | 88.0 (67.4, 100.0) | 87.4 (67.4, 100.0) | | 87.8 (67.4, 100.0) | 87.3 (67.4, 100.0) | |
| Non-event deaths during follow-up, sum, among all users | | | 1,131 | 927 | | 810 | 827 | |
| **Demographics** | | Group | % (unless otherwise noted) | | SDif* | % (unless otherwise noted) | | SDif* |
| Age in years at cohort entry, continuous | | Median  (Q1-Q3) | 58.5 (49.0-67.2) | 59.1 (49.2-67.5) | 0.03 | 58.7 (48.9-67.4) | 58.9 (49.1-67.5) | 0.01 |
| Sex | | Female | 61.5 | 63.2 | 0.03 | 62.6 | 62.8 | 0.00 |
| Race | | White | 34.7 | 35.3 | 0.01 | 35.8 | 35.4 | 0.01 |
|  |  | Black | 16.3 | 17.6 | 0.04 | 17.2 | 17.3 | 0.00 |
|  |  | Hispanic/Latino | 25.0 | 21.3 | 0.09 | 22.1 | 22.0 | 0.00 |
|  |  | Other/Unknown | 24.1 | 25.8 | 0.04 | 24.9 | 25.3 | 0.01 |
| State of residence | | CA | 49.7 | 43.4 | 0.13 | 45.7 | 44.9 | 0.02 |
|  |  | FL | 9.4 | 11.4 | 0.06 | 10.7 | 10.9 | 0.01 |
|  |  | NY | 26.1 | 31.4 | 0.12 | 28.8 | 29.9 | 0.02 |
|  |  | OH | 9.1 | 8.0 | 0.04 | 8.7 | 8.4 | 0.01 |
|  |  | PA | 5.6 | 5.8 | 0.01 | 6.1 | 5.9 | 0.01 |
| Calendar year of cohort entry^§^ | | 2000 | 6.0 | 11.1 | 0.18 | 8.8 | 10.4 | 0.05 |
|  |  | 2001 | 7.3 | 11.8 | 0.16 | 10.1 | 11.3 | 0.04 |
|  |  | 2002 | 7.4 | 11.1 | 0.13 | 10.1 | 10.8 | 0.02 |
|  |  | 2003 | 8.0 | 12.8 | 0.16 | 10.7 | 12.5 | 0.06 |
|  |  | 2004 | 7.0 | 12.7 | 0.19 | 8.9 | 12.7 | 0.12 |
|  |  | 2005 | 9.0 | 13.3 | 0.14 | 10.7 | 13.5 | 0.09 |
|  |  | 2006 | 10.5 | 15.1 | 0.14 | 11.3 | 15.8 | 0.13 |
|  |  | 2007 | 8.4 | 7.0 | 0.05 | 7.7 | 7.5 | 0.01 |
|  |  | 2008 | 7.7 | 2.1 | 0.26 | 5.8 | 2.2 | 0.18 |
|  |  | 2009 | 9.1 | 2.0 | 0.31 | 5.7 | 2.2 | 0.18 |
|  |  | 2010 | 9.9 | 1.0 | 0.40 | 5.6 | 1.0 | 0.26 |
|  |  | 2011 | 6.3 | 0.1 | 0.36 | 3.2 | 0.1 | 0.25 |
|  |  | 2012 | 3.4 | 0.0 | 0.27 | 1.6 | 0.0 | 0.18 |
| Medicare enrolled | | Yes | 49.3 | 51.0 | 0.03 | 49.7 | 50.3 | 0.01 |
| Nursing home residence ever during baseline | | Yes | 4.7 | 5.2 | 0.02 | 5.0 | 5.0 | 0.00 |
| **Healthcare use intensity measures, in baseline period**** | | Group | Measure of central tendency | | SDif* | Measure of central tendency | | SDif* |
| # prescriptions dispensed, total | | Median  (Q1-Q3) | 51.0 (23.0-88.0) | 50.0 (22.0-86.0) | 0.03 | 49.0 (21.0-85.0) | 50.0 (21.0-86.0) | 0.02 |
| # prescriptions dispensed, by unique drug | |  | 14.0 (8.0-20.0) | 14.0 (8.0-21.0) | 0.01 | 13.0 (7.0-20.0) | 13.0 (8.0-20.0) | 0.02 |
| # outpatient diagnosis codes, total | |  | 33.0 (15.0-72.0) | 36.0 (16.0-75.0) | 0.03 | 34.0 (15.0-72.0) | 35.0 (16.0-73.0) | 0.02 |
| # outpatient diagnosis codes, by unique code | |  | 13.0 (6.0-22.0) | 13.0 (7.0-22.0) | 0.04 | 13.0 (7.0-22.0) | 13.0 (7.0-22.0) | 0.02 |
| # outpatient CPT / HCPCS procedure codes, total | |  | 41.0 (17.0-83.0) | 41.0 (19.0-83.0) | 0.00 | 40.0 (18.0-80.0) | 41.0 (18.0-82.0) | 0.01 |
| # outpatient CPT / HCPCS procedure codes, by unique code | |  | 24.0 (12.0-42.0) | 24.0 (13.0-42.0) | 0.01 | 24.0 (12.0-41.0) | 24.0 (12.0-42.0) | 0.01 |
| **Other investigator pre-defined covariates, in baseline period** | | Group | % | | SDif* | % | | SDif* |
| Disorders of lipid metabolism | | Yes | 50.5 | 49.8 | 0.01 | 49.2 | 49.6 | 0.01 |
| Rheumatic heart disease, chronic | |  | 2.2 | 2.7 | 0.03 | 2.5 | 2.6 | 0.01 |
| Hypertensive disease | |  | 64.0 | 67.0 | 0.06 | 65.3 | 66.0 | 0.01 |
| Ischemic heart disease | |  | 22.1 | 25.1 | 0.07 | 23.6 | 24.2 | 0.01 |
| Conduction disorders | |  | 1.8 | 2.0 | 0.02 | 1.8 | 1.9 | 0.01 |
| Heart failure / cardiomyopathy | |  | 11.6 | 13.4 | 0.05 | 12.6 | 12.8 | 0.01 |
| Cardiomegaly | |  | 5.4 | 6.1 | 0.03 | 5.7 | 5.9 | 0.01 |
| Congenital anomalies of the heart, other | |  | 1.5 | 1.8 | 0.02 | 1.6 | 1.7 | 0.00 |
| ICD / pacemaker use | |  | 0.8 | 0.7 | 0.01 | 0.7 | 0.7 | 0.00 |
| Kidney disease | |  | 17.1 | 16.7 | 0.01 | 16.1 | 16.5 | 0.01 |
| Liver disease | |  | 11.3 | 11.2 | 0.00 | 10.9 | 11.1 | 0.01 |
| Osteoporosis | |  | 6.0 | 6.2 | 0.01 | 6.0 | 6.0 | 0.00 |
| Depression | |  | 26.2 | 25.8 | 0.01 | 25.4 | 25.6 | 0.01 |
| Obesity | |  | 11.3 | 10.9 | 0.01 | 10.7 | 10.8 | 0.00 |
| Tobacco use | |  | 7.5 | 6.7 | 0.03 | 6.7 | 6.8 | 0.00 |
| Alcohol abuse | |  | 2.5 | 2.9 | 0.02 | 2.8 | 2.8 | 0.00 |
| Hypoglycemia, serious | |  | 2.7 | 2.9 | 0.01 | 2.8 | 2.8 | 0.00 |
| Diabetes mellitus, type 2^‡^ | |  | 93.7 | 92.3 | 0.05 | 92.5 | 92.5 | 0.00 |
| Adapted Diabetes Complications Severity Index | | 0 | 44.4 | 41.1 | 0.07 | 42.7 | 42.1 | 0.01 |
|  |  | 1 | 14.6 | 15.1 | 0.01 | 15.0 | 15.0 | 0.00 |
|  |  | 2 | 15.5 | 16.2 | 0.02 | 16.1 | 16.0 | 0.00 |
|  |  | 3 | 8.2 | 8.6 | 0.02 | 8.4 | 8.5 | 0.01 |
|  |  | 4 | 6.4 | 6.9 | 0.02 | 6.7 | 6.8 | 0.00 |
|  |  | 5+ | 10.9 | 12.1 | 0.04 | 11.2 | 11.6 | 0.01 |
| **Other investigator pre-defined covariates, 30 days prior to cohort entry**^†^ | | Group | % | | SDif* | % | | SDif* |
| Antidiabetic drug | alpha-glucosidase inhibitor | Yes | 0.5 | 0.6 | 0.01 | 0.6 | 0.6 | 0.00 |
|  | amylin analog | Yes | 0.0 | 0.0 | 0.01 | 0.0 | 0.0 | 0.00 |
|  | dipeptidyl peptidase-4 inhibitor | Yes | 2.3 | 0.2 | 0.19 | 0.2 | 0.2 | 0.00 |
|  | glucagonlike peptide 1 receptor agonist | Yes | 0.3 | 0.1 | 0.05 | 0.1 | 0.1 | 0.01 |
|  | insulin | Yes | 11.7 | 11.2 | 0.02 | 11.1 | 11.1 | 0.00 |
|  | metformin | Yes | 31.3 | 28.7 | 0.06 | 28.5 | 28.9 | 0.01 |
|  | none^\|^ | Yes | 50.3 | 52.0 | 0.04 | 52.7 | 52.2 | 0.01 |
|  | meglitinide | Yes | 1.3 | 1.3 | 0.01 | 1.3 | 1.3 | 0.00 |
|  | sodium-glucose co-transporter 2 inhibitor^§§^ | Yes | — | — | — | — | — | — |
|  | sulfonylurea | Yes | 26.4 | 27.1 | 0.02 | 26.5 | 26.8 | 0.01 |
| CYP2C9 inhibitor | | Yes | 4.8 | 5.2 | 0.02 | 5.1 | 5.2 | 0.00 |
| CYP3A4 inhibitor | | Yes | 4.8 | 5.8 | 0.04 | 5.5 | 5.5 | 0.00 |
| CYP2C8 inhibitor | | Yes | 4.5 | 4.0 | 0.02 | 4.0 | 4.0 | 0.00 |
| CYP2C9 inducer | | Yes | 0.9 | 1.0 | 0.01 | 1.0 | 1.0 | 0.00 |
| CYP3A4 inducer | | Yes | 1.7 | 1.9 | 0.02 | 1.9 | 1.9 | 0.00 |
| Drug with known risk of torsade de pointes^ǁ^ | | Yes | 9.0 | 8.4 | 0.02 | 8.4 | 8.4 | 0.00 |
| Drug with known, possible, or conditional risk of torsade de pointes^ǁ^ | | Yes | 49.3 | 47.7 | 0.03 | 47.2 | 47.6 | 0.01 |
| ≥ 5 prescription dispensings for unique drugs, a potential indicator of polypharmacy | | Yes | 48.0 | 46.5 | 0.03 | 45.6 | 46.2 | 0.01 |

CA = California; CPT = Current Procedural Terminology; CYP = hepatic cytochrome P450; FL = Florida; HCPCS = Healthcare Common Procedure Coding System; ICD = implantable cardioverter-defibrillator; NY = New York; OH = Ohio; PA = Pennsylvania; Q = quartile; PDC = proportion of days covered; PS = propensity score; SDif = standardized difference

* Standardized difference versus pioglitazone; a pictorial representation of covariate balance is presented in **Additional file 1. Figure S1**.

** The following healthcare utilization covariates were excluded from presentation in the table, as their median values were zero for each thiazolidinedione: # inpatient ICD-9 diagnosis codes; # unique inpatient ICD-9 diagnosis codes; # inpatient ICD-9 procedure codes; # unique inpatient ICD-9 procedure codes; # inpatient CPT/HCPCS procedure codes; # unique inpatient CPT/HCPCS procedure codes; # outpatient ICD-9 procedure codes; # unique outpatient ICD-9 procedure codes; # other setting ICD-9 diagnosis codes; # unique other setting ICD-9 diagnosis codes; # other setting ICD-9 procedure codes; # unique other setting ICD-9 procedure codes

† Antimicrobial drugs in each category were examined within 14 (rather than 30) days prior to cohort entry; these agents are typically prescribed for acute conditions (e.g., sulfamethoxazole within CYP2C9 inhibitors, erythromycin within CYP3A4 inhibitors, drugs with known risk of torsade de pointes, and drugs with known, possible, or conditional risk of torsade de pointes)

‡ Defined by ratio of type 1 (ICD-9 250.X1 or 250.X3) to type 2 (ICD-9 250.X0 or 250.X2) codes ≤0.5, ascertained during baseline and on cohort entry date

§ Prespecified covariate not forced into propensity score, but included as a categorical variable in outcome model

§§ Not marketed during years of study

| Postspecified covariate not forced into propensity score

ǁ Per CredibleMeds (AZCERT Inc.: Oro Valley, AZ)

**Additional file 1. Table S6. Covariates empirically identified by the high-dimensional propensity score method**

| **Data dimension** | **Variable** | **Code** | **Code description** | **Z-bias = 5*** |
| --- | --- | --- | --- | --- |
| Drug | D01V142Freq | Drug-specific NDCs | albuterol |  |
| Drug | D01V142Once | Drug-specific NDCs | albuterol |  |
| Drug | D01V142Spor | Drug-specific NDCs | albuterol |  |
| Drug | D01V784Once | Drug-specific NDCs | atorvastatin | Y |
| Drug | D01V784Spor | Drug-specific NDCs | atorvastatin | Y |
| Drug | D01V785Once | Drug-specific NDCs | azithromycin |  |
| Drug | D01V176Once | Drug-specific NDCs | calcium carbonate |  |
| Drug | D01V176Spor | Drug-specific NDCs | calcium carbonate |  |
| Drug | D01V015Once | Drug-specific NDCs | calcium carbonate |  |
| Drug | D01V062Once | Drug-specific NDCs | captopril |  |
| Drug | D01V255Freq | Drug-specific NDCs | carvedilol |  |
| Drug | D01V255Once | Drug-specific NDCs | carvedilol |  |
| Drug | D01V255Spor | Drug-specific NDCs | carvedilol |  |
| Drug | D01V462Once | Drug-specific NDCs | celecoxib | Y |
| Drug | D01V779Once | Drug-specific NDCs | cetirizine hydrochloride |  |
| Drug | D01V645Once | Drug-specific NDCs | citalopram |  |
| Drug | D01V1045Freq | Drug-specific NDCs | clopidogrel bisulfate |  |
| Drug | D01V1045Once | Drug-specific NDCs | clopidogrel bisulfate |  |
| Drug | D01V1045Spor | Drug-specific NDCs | clopidogrel bisulfate |  |
| Drug | D01V477Once | Drug-specific NDCs | clotrimazole | Y |
| Drug | D01V289Freq | Drug-specific NDCs | codeine phosphate |  |
| Drug | D01V289Once | Drug-specific NDCs | codeine phosphate | Y |
| Drug | D01V586Once | Drug-specific NDCs | conjugated estrogens | Y |
| Drug | D01V224Once | Drug-specific NDCs | cyclobenzaprine hydrochloride |  |
| Drug | D01V644Freq | Drug-specific NDCs | digoxin |  |
| Drug | D01V644Once | Drug-specific NDCs | digoxin |  |
| Drug | D01V644Spor | Drug-specific NDCs | digoxin |  |
| Drug | D01V089Once | Drug-specific NDCs | diltiazem hydrochloride |  |
| Drug | D01V089Spor | Drug-specific NDCs | diltiazem hydrochloride |  |
| Drug | D01V860Freq | Drug-specific NDCs | divalproex sodium |  |
| Drug | D01V860Once | Drug-specific NDCs | divalproex sodium |  |
| Drug | D01V860Spor | Drug-specific NDCs | divalproex sodium |  |
| Drug | D01V187Freq | Drug-specific NDCs | enalapril maleate |  |
| Drug | D01V187Once | Drug-specific NDCs | enalapril maleate |  |
| Drug | D01V187Spor | Drug-specific NDCs | enalapril maleate |  |
| Drug | D01V1036Once | Drug-specific NDCs | escitalopram oxalate |  |
| Drug | D01V1176Once | Drug-specific NDCs | esomeprazole | Y |
| Drug | D01V1176Spor | Drug-specific NDCs | esomeprazole | Y |
| Drug | D01V1358Freq | Drug-specific NDCs | ezetimibe |  |
| Drug | D01V1358Once | Drug-specific NDCs | ezetimibe |  |
| Drug | D01V1358Spor | Drug-specific NDCs | ezetimibe |  |
| Drug | D01V225Once | Drug-specific NDCs | famotidine |  |
| Drug | D01V225Spor | Drug-specific NDCs | famotidine |  |
| Drug | D01V851Once | Drug-specific NDCs | fenofibrate |  |
| Drug | D01V995Once | Drug-specific NDCs | fexofenadine hydrochloride |  |
| Drug | D01V981Once | Drug-specific NDCs | fosinopril sodium | Y |
| Drug | D01V143Freq | Drug-specific NDCs | furosemide |  |
| Drug | D01V143Once | Drug-specific NDCs | furosemide |  |
| Drug | D01V143Spor | Drug-specific NDCs | furosemide |  |
| Drug | D01V803Freq | Drug-specific NDCs | gabapentin |  |
| Drug | D01V803Once | Drug-specific NDCs | gabapentin | Y |
| Drug | D01V803Spor | Drug-specific NDCs | gabapentin | Y |
| Drug | D01V063Once | Drug-specific NDCs | hydrochlorothiazide | Y |
| Drug | D01V429Once | Drug-specific NDCs | hydrocodone bitartrate | Y |
| Drug | D01V331Once | Drug-specific NDCs | ibuprofen |  |
| Drug | D01V1094Once | Drug-specific NDCs | insulin aspart |  |
| Drug | D01V1094Spor | Drug-specific NDCs | insulin aspart |  |
| Drug | D01V036Once | Drug-specific NDCs | insulin lispro |  |
| Drug | D01V036Spor | Drug-specific NDCs | insulin lispro |  |
| Drug | D01V044Freq | Drug-specific NDCs | insulin nph human recombinant |  |
| Drug | D01V044Once | Drug-specific NDCs | insulin nph human recombinant |  |
| Drug | D01V044Spor | Drug-specific NDCs | insulin nph human recombinant |  |
| Drug | D01V041Freq | Drug-specific NDCs | insulin regular human recombinant |  |
| Drug | D01V041Once | Drug-specific NDCs | insulin regular human recombinant |  |
| Drug | D01V041Spor | Drug-specific NDCs | insulin regular human recombinant |  |
| Drug | D01V641Freq | Drug-specific NDCs | ipratropium bromide |  |
| Drug | D01V641Once | Drug-specific NDCs | ipratropium bromide |  |
| Drug | D01V641Spor | Drug-specific NDCs | ipratropium bromide |  |
| Drug | D01V302Freq | Drug-specific NDCs | isosorbide dinitrate |  |
| Drug | D01V302Once | Drug-specific NDCs | isosorbide dinitrate |  |
| Drug | D01V302Spor | Drug-specific NDCs | isosorbide dinitrate |  |
| Drug | D01V292Once | Drug-specific NDCs | isosorbide mononitrate |  |
| Drug | D01V292Spor | Drug-specific NDCs | isosorbide mononitrate |  |
| Drug | D01V764Once | Drug-specific NDCs | lansoprazole |  |
| Drug | D01V764Spor | Drug-specific NDCs | lansoprazole |  |
| Drug | D01V566Once | Drug-specific NDCs | levofloxacin |  |
| Drug | D01V592Once | Drug-specific NDCs | levothyroxine sodium | Y |
| Drug | D01V188Freq | Drug-specific NDCs | lisinopril |  |
| Drug | D01V188Once | Drug-specific NDCs | lisinopril | Y |
| Drug | D01V188Spor | Drug-specific NDCs | lisinopril |  |
| Drug | D01V755Once | Drug-specific NDCs | loratadine |  |
| Drug | D01V755Spor | Drug-specific NDCs | loratadine |  |
| Drug | D01V657Once | Drug-specific NDCs | meloxicam |  |
| Drug | D01V087Once | Drug-specific NDCs | metformin hydrochloride |  |
| Drug | D01V087Spor | Drug-specific NDCs | metformin hydrochloride | Y |
| Drug | D01V492Freq | Drug-specific NDCs | metoprolol tartrate |  |
| Drug | D01V492Once | Drug-specific NDCs | metoprolol tartrate |  |
| Drug | D01V492Spor | Drug-specific NDCs | metoprolol tartrate |  |
| Drug | D01V335Freq | Drug-specific NDCs | multiple vitamins |  |
| Drug | D01V092Once | Drug-specific NDCs | naproxen |  |
| Drug | D01V487Once | Drug-specific NDCs | nifedipine |  |
| Drug | D01V597Freq | Drug-specific NDCs | nitroglycerin |  |
| Drug | D01V597Once | Drug-specific NDCs | nitroglycerin |  |
| Drug | D01V024Freq | Drug-specific NDCs | olanzapine |  |
| Drug | D01V024Once | Drug-specific NDCs | olanzapine |  |
| Drug | D01V024Spor | Drug-specific NDCs | olanzapine |  |
| Drug | D01V1033Freq | Drug-specific NDCs | omeprazole | Y |
| Drug | D01V1033Once | Drug-specific NDCs | omeprazole | Y |
| Drug | D01V1033Spor | Drug-specific NDCs | omeprazole | Y |
| Drug | D01V575Once | Drug-specific NDCs | oxycodone hydrochloride | Y |
| Drug | D01V575Spor | Drug-specific NDCs | oxycodone hydrochloride |  |
| Drug | D01V290Once | Drug-specific NDCs | pantoprazole |  |
| Drug | D01V500Once | Drug-specific NDCs | paroxetine hydrochloride | Y |
| Drug | D01V510Once | Drug-specific NDCs | potassium chloride |  |
| Drug | D01V510Spor | Drug-specific NDCs | potassium chloride |  |
| Drug | D01V350Once | Drug-specific NDCs | pseudoephedrine hydrochloride |  |
| Drug | D01V656Spor | Drug-specific NDCs | quetiapine fumarate |  |
| Drug | D01V798Once | Drug-specific NDCs | quinapril hydrochloride |  |
| Drug | D01V798Spor | Drug-specific NDCs | quinapril hydrochloride |  |
| Drug | D01V774Once | Drug-specific NDCs | quinine sulfate |  |
| Drug | D01V646Once | Drug-specific NDCs | risperidone |  |
| Drug | D01V646Spor | Drug-specific NDCs | risperidone |  |
| Drug | D01V199Spor | Drug-specific NDCs | rofecoxib | Y |
| Drug | D01V1211Once | Drug-specific NDCs | rosuvastatin calcium | Y |
| Drug | D01V212Once | Drug-specific NDCs | simvastatin | Y |
| Drug | D01V202Freq | Drug-specific NDCs | sitagliptin |  |
| Drug | D01V202Once | Drug-specific NDCs | sitagliptin |  |
| Drug | D01V202Spor | Drug-specific NDCs | sitagliptin |  |
| Drug | D01V013Freq | Drug-specific NDCs | sodium chloride |  |
| Drug | D01V013Once | Drug-specific NDCs | sodium chloride |  |
| Drug | D01V458Freq | Drug-specific NDCs | spironolactone |  |
| Drug | D01V708Once | Drug-specific NDCs | terconazole |  |
| Drug | D01V534Once | Drug-specific NDCs | theophylline |  |
| Drug | D01V166Once | Drug-specific NDCs | triamterene |  |
| Drug | D01V901Freq | Drug-specific NDCs | valsartan |  |
| Drug | D01V901Once | Drug-specific NDCs | valsartan |  |
| Drug | D01V901Spor | Drug-specific NDCs | valsartan |  |
| Drug | D01V139Spor | Drug-specific NDCs | verapamil hydrochloride |  |
| Drug | D01V177Once | Drug-specific NDCs | vitamin D |  |
| Drug | D01V177Spor | Drug-specific NDCs | vitamin D |  |
| Drug | D01V688Once | Drug-specific NDCs | warfarin sodium |  |
| Inpatient CPT Px | D04V298Once | 94657 | ventilation assist and management, initiation of pressure or volume preset ventilators for assisted or controlled breathing; subsequent days |  |
| Inpatient ICD-9 Dx | D02V085Once | 410 | acute myocardial infarct |  |
| Inpatient ICD-9 Dx | D02V063Once | 584 | acute renal failure |  |
| Inpatient ICD-9 Dx | D02V031Once | 285 | anemia nec/nos |  |
| Inpatient ICD-9 Dx | D02V113Once | 413 | angina pectoris |  |
| Inpatient ICD-9 Dx | D02V051Freq | 427 | cardiac dysrhythmias |  |
| Inpatient ICD-9 Dx | D02V051Once | 427 | cardiac dysrhythmias |  |
| Inpatient ICD-9 Dx | D02V050Once | 425 | cardiomyopathy |  |
| Inpatient ICD-9 Dx | D02V068Once | 995 | certain adverse eff nec |  |
| Inpatient ICD-9 Dx | D02V027Freq | 496 | chr airway obstruct nec |  |
| Inpatient ICD-9 Dx | D02V027Once | 496 | chr airway obstruct nec |  |
| Inpatient ICD-9 Dx | D02V053Once | 491 | chronic bronchitis |  |
| Inpatient ICD-9 Dx | D02V028Freq | 585 | chronic renal failure |  |
| Inpatient ICD-9 Dx | D02V028Once | 585 | chronic renal failure |  |
| Inpatient ICD-9 Dx | D02V009Once | 707 | chronic ulcer of skin |  |
| Inpatient ICD-9 Dx | D02V001Freq | 250 | diabetes mellitus |  |
| Inpatient ICD-9 Dx | D02V001Once | 250 | diabetes mellitus |  |
| Inpatient ICD-9 Dx | D02V030Once | V58 | encountr proc/aftrcr nec |  |
| Inpatient ICD-9 Dx | D02V226Once | 345 | epilepsy |  |
| Inpatient ICD-9 Dx | D02V008Freq | 401 | essential hypertension |  |
| Inpatient ICD-9 Dx | D02V008Once | 401 | essential hypertension |  |
| Inpatient ICD-9 Dx | D02V002Once | 276 | fluid/electrolyte dis |  |
| Inpatient ICD-9 Dx | D02V188Once | 578 | gastrointestinal hemorr |  |
| Inpatient ICD-9 Dx | D02V016Once | 780 | general symptoms |  |
| Inpatient ICD-9 Dx | D02V025Freq | 428 | heart failure |  |
| Inpatient ICD-9 Dx | D02V025Once | 428 | heart failure |  |
| Inpatient ICD-9 Dx | D02V099Once | V12 | hx of disease nec |  |
| Inpatient ICD-9 Dx | D02V263Once | 404 | hyperten heart/renal dis |  |
| Inpatient ICD-9 Dx | D02V023Freq | 403 | hypertensive renal dis |  |
| Inpatient ICD-9 Dx | D02V119Once | 438 | late eff cerebrovasc dis |  |
| Inpatient ICD-9 Dx | D02V243Once | 583 | nephritis nos |  |
| Inpatient ICD-9 Dx | D02V376Once | 581 | nephrotic syndrome |  |
| Inpatient ICD-9 Dx | D02V093Once | 412 | old myocardial infarct |  |
| Inpatient ICD-9 Dx | D02V087Once | 411 | oth ac ischemic hrt dis |  |
| Inpatient ICD-9 Dx | D02V048Freq | 414 | oth chr ischemic hrt dis |  |
| Inpatient ICD-9 Dx | D02V048Once | 414 | oth chr ischemic hrt dis |  |
| Inpatient ICD-9 Dx | D02V024Once | 424 | oth endocardial disease |  |
| Inpatient ICD-9 Dx | D02V011Once | 443 | oth periph vascular dis |  |
| Inpatient ICD-9 Dx | D02V057Freq | V45 | oth postsurgical states |  |
| Inpatient ICD-9 Dx | D02V057Once | V45 | oth postsurgical states |  |
| Inpatient ICD-9 Dx | D02V019Freq | V62 | oth psychosocial circum |  |
| Inpatient ICD-9 Dx | D02V132Once | 593 | oth renal & ureteral dis |  |
| Inpatient ICD-9 Dx | D02V005Once | 599 | oth urinary tract disor |  |
| Inpatient ICD-9 Dx | D02V238Once | 348 | other brain conditions |  |
| Inpatient ICD-9 Dx | D02V182Once | 518 | other lung diseases |  |
| Inpatient ICD-9 Dx | D02V143Once | E849 | place of occurrence |  |
| Inpatient ICD-9 Dx | D02V026Once | 486 | pneumonia |  |
| Inpatient ICD-9 Dx | D02V163Once | V57 | rehabilitation procedure |  |
| Inpatient ICD-9 Dx | D02V101Once | 996 | replace & graft complic |  |
| Inpatient ICD-9 Dx | D02V029Once | 786 | resp sys/oth chest symp |  |
| Inpatient ICD-9 Dx | D02V010Once | 362 | retinal disorders nec |  |
| Inpatient ICD-9 Dx | D02V034Once | 038 | septicemia |  |
| Inpatient ICD-9 Dx | D02V211Once | V85 | body mass index |  |
| Inpatient ICD-9 Px | D03V003Once | 89 | interview/consult/exam |  |
| Inpatient ICD-9 Px | D03V000Freq | 45 | intest incis/excis/anast |  |
| Inpatient ICD-9 Px | D03V000Once | 45 | intest incis/excis/anast |  |
| Inpatient ICD-9 Px | D03V008Once | 96 | non-op intubat & irrigat |  |
| Inpatient ICD-9 Px | D03V017Once | 36 | ops on heart vessels |  |
| Inpatient ICD-9 Px | D03V017Spor | 36 | ops on heart vessels |  |
| Inpatient ICD-9 Px | D03V002Once | 88 | other dx radiology |  |
| Inpatient ICD-9 Px | D03V002Spor | 88 | other dx radiology |  |
| Inpatient ICD-9 Px | D03V010Once | 37 | other heart/pericard ops |  |
| Inpatient ICD-9 Px | D03V006Freq | 99 | other nonoperative proc |  |
| Inpatient ICD-9 Px | D03V006Once | 99 | other nonoperative proc |  |
| Inpatient ICD-9 Px | D03V028Freq | 39 | other ops on vessels |  |
| Inpatient ICD-9 Px | D03V028Once | 39 | other ops on vessels |  |
| Inpatient ICD-9 Px | D03V019Freq | 93 | pt |  |
| Inpatient ICD-9 Px | D03V019Once | 93 | pt |  |
| Other Setting ICD-9 Dx | D08V045Once | 436 | cva |  |
| Other Setting ICD-9 Dx | D08V000Once | 250 | diabetes mellitus |  |
| Other Setting ICD-9 Dx | D08V044Once | 728 | dis of muscle/lig/fascia |  |
| Other Setting ICD-9 Dx | D08V004Freq | 780 | general symptoms |  |
| Other Setting ICD-9 Dx | D08V048Once | 428 | heart failure |  |
| Other Setting ICD-9 Dx | D08V127Once | V15 | oth hx of health hazards |  |
| Other Setting ICD-9 Dx | D08V037Once | 443 | oth periph vascular dis |  |
| Other Setting ICD-9 Dx | D08V214Freq | 288 | wbc disorders |  |
| Other Setting ICD-9 Dx | D08V214Once | 288 | wbc disorders |  |
| Outpatient CPT Px | D07V100Once | 76092 | mammography, screening, bilateral |  |
| Outpatient CPT Px | D07V082Freq | 80054 | comprehensive metabolic panel | Y |
| Outpatient CPT Px | D07V082Once | 80054 | comprehensive metabolic panel |  |
| Outpatient CPT Px | D07V047Freq | 85024 | blood count; hemogram and platelet count | Y |
| Outpatient CPT Px | D07V047Once | 85024 | blood count; hemogram and platelet count |  |
| Outpatient CPT Px | D07V166Once | 90659 | influenza virus vaccine, whole virus |  |
| Outpatient CPT Px | D07V181Freq | A0425 | ambulance; ground mileage |  |
| Outpatient CPT Px | D07V181Once | A0425 | ambulance; ground mileage |  |
| Outpatient CPT Px | D07V039Freq | A0427 | ambulance service, advanced life support, emergency transport, level 1 |  |
| Outpatient CPT Px | D07V039Once | A0427 | ambulance service, advanced life support, emergency transport, level 1 |  |
| Outpatient CPT Px | D07V544Once | 84075 | assay alkaline phosphatase |  |
| Outpatient CPT Px | D07V316Once | 82465 | assay bld/serum cholesterol |  |
| Outpatient CPT Px | D07V336Freq | 82435 | assay blood chloride |  |
| Outpatient CPT Px | D07V336Once | 82435 | assay blood chloride |  |
| Outpatient CPT Px | D07V384Freq | 82310 | assay calcium |  |
| Outpatient CPT Px | D07V384Once | 82310 | assay calcium |  |
| Outpatient CPT Px | D07V280Once | 82565 | assay creatinine |  |
| Outpatient CPT Px | D07V107Freq | 82728 | assay ferritin |  |
| Outpatient CPT Px | D07V107Once | 82728 | assay ferritin |  |
| Outpatient CPT Px | D07V337Freq | 82947 | assay glucose, blood quant |  |
| Outpatient CPT Px | D07V337Once | 82947 | assay glucose, blood quant |  |
| Outpatient CPT Px | D07V722Freq | 83718 | assay lipoprotein |  |
| Outpatient CPT Px | D07V722Once | 83718 | assay lipoprotein |  |
| Outpatient CPT Px | D07V114Freq | 83735 | assay magnesium |  |
| Outpatient CPT Px | D07V114Once | 83735 | assay magnesium |  |
| Outpatient CPT Px | D07V293Once | 84100 | assay phosphorus |  |
| Outpatient CPT Px | D07V457Once | 84155 | assay protein, serum |  |
| Outpatient CPT Px | D07V083Once | 84153 | assay psa, total | Y |
| Outpatient CPT Px | D07V541Freq | 82040 | assay serum albumin |  |
| Outpatient CPT Px | D07V541Once | 82040 | assay serum albumin |  |
| Outpatient CPT Px | D07V234Freq | 84132 | assay serum potassium |  |
| Outpatient CPT Px | D07V234Once | 84132 | assay serum potassium |  |
| Outpatient CPT Px | D07V339Freq | 84295 | assay serum sodium |  |
| Outpatient CPT Px | D07V339Once | 84295 | assay serum sodium |  |
| Outpatient CPT Px | D07V318Once | 84478 | assay triglycerides |  |
| Outpatient CPT Px | D07V046Freq | 84484 | assay troponin, quant |  |
| Outpatient CPT Px | D07V046Once | 84484 | assay troponin, quant |  |
| Outpatient CPT Px | D07V340Freq | 84520 | assay urea nitrogen |  |
| Outpatient CPT Px | D07V340Once | 84520 | assay urea nitrogen |  |
| Outpatient CPT Px | D07V231Once | 82570 | assay urine creatinine | Y |
| Outpatient CPT Px | D07V542Once | 82247 | bilirubin, total |  |
| Outpatient CPT Px | D07V320Once | 87040 | blood culture for bacteria |  |
| Outpatient CPT Px | D07V239Once | 93016 | cardiovascular stress test |  |
| Outpatient CPT Px | D07V240Once | 93018 | cardiovascular stress test |  |
| Outpatient CPT Px | D07V024Freq | 71020 | chest x-ray |  |
| Outpatient CPT Px | D07V024Once | 71020 | chest x-ray |  |
| Outpatient CPT Px | D07V043Freq | 71010 | chest x-ray |  |
| Outpatient CPT Px | D07V043Once | 71010 | chest x-ray |  |
| Outpatient CPT Px | D07V008Freq | 85025 | complete cbc w/auto diff wbc |  |
| Outpatient CPT Px | D07V008Once | 85025 | complete cbc w/auto diff wbc |  |
| Outpatient CPT Px | D07V008Spor | 85025 | complete cbc w/auto diff wbc |  |
| Outpatient CPT Px | D07V211Freq | 85027 | complete cbc, automated |  |
| Outpatient CPT Px | D07V211Once | 85027 | complete cbc, automated |  |
| Outpatient CPT Px | D07V025Freq | 80053 | comprehen metabolic panel | Y |
| Outpatient CPT Px | D07V025Once | 80053 | comprehen metabolic panel | Y |
| Outpatient CPT Px | D07V025Spor | 80053 | comprehen metabolic panel | Y |
| Outpatient CPT Px | D07V230Once | 82553 | creatine, mb fraction |  |
| Outpatient CPT Px | D07V575Freq | 99291 | critical care, first hour |  |
| Outpatient CPT Px | D07V575Once | 99291 | critical care, first hour |  |
| Outpatient CPT Px | D07V028Once | 87077 | culture aerobic identify |  |
| Outpatient CPT Px | D07V067Once | D0110 | dental; initial oral examination |  |
| Outpatient CPT Px | D07V015Once | D1110 | dental; prophylaxis | Y |
| Outpatient CPT Px | D07V150Once | 11721 | debride nail, 6 or more |  |
| Outpatient CPT Px | D07V150Spor | 11721 | debride nail, 6 or more |  |
| Outpatient CPT Px | D07V135Once | 93325 | doppler color flow add-on |  |
| Outpatient CPT Px | D07V134Once | 93320 | doppler echo exam heart |  |
| Outpatient CPT Px | D07V168Freq | 93000 | ecg complete |  |
| Outpatient CPT Px | D07V031Freq | 93010 | ecg report |  |
| Outpatient CPT Px | D07V031Once | 93010 | ecg report |  |
| Outpatient CPT Px | D07V053Once | 93005 | ecg tracing |  |
| Outpatient CPT Px | D07V133Once | 93307 | echo exam heart |  |
| Outpatient CPT Px | D07V833Once | 80051 | electrolyte panel |  |
| Outpatient CPT Px | D07V034Freq | 99285 | emergency dept visit |  |
| Outpatient CPT Px | D07V034Once | 99285 | emergency dept visit |  |
| Outpatient CPT Px | D07V086Once | 90658 | flu vaccine, 3 yrs & >, im | Y |
| Outpatient CPT Px | D07V016Freq | G0001 | routine venipuncture for collection of specimen |  |
| Outpatient CPT Px | D07V016Once | G0001 | routine venipuncture for collection of specimen |  |
| Outpatient CPT Px | D07V016Spor | G0001 | routine venipuncture for collection of specimen |  |
| Outpatient CPT Px | D07V232Once | 82962 | glucose blood test |  |
| Outpatient CPT Px | D07V232Spor | 82962 | glucose blood test |  |
| Outpatient CPT Px | D07V006Freq | 83036 | glycosylated hemoglobin test |  |
| Outpatient CPT Px | D07V006Spor | 83036 | glycosylated hemoglobin test |  |
| Outpatient CPT Px | D07V381Once | 78480 | heart function add-on |  |
| Outpatient CPT Px | D07V379Once | 78465 | heart image (3d), multiple |  |
| Outpatient CPT Px | D07V380Once | 78478 | heart wall motion add-on |  |
| Outpatient CPT Px | D07V004Freq | 80076 | hepatic function panel |  |
| Outpatient CPT Px | D07V004Once | 80076 | hepatic function panel | Y |
| Outpatient CPT Px | D07V130Freq | 87340 | hepatitis b surface ag, eia |  |
| Outpatient CPT Px | D07V032Freq | 99238 | hospital discharge day |  |
| Outpatient CPT Px | D07V032Once | 99238 | hospital discharge day |  |
| Outpatient CPT Px | D07V250Once | 99239 | hospital discharge day |  |
| Outpatient CPT Px | D07V687Once | 93556 | imaging, cardiac cath |  |
| Outpatient CPT Px | D07V085Once | 90471 | immunization admin |  |
| Outpatient CPT Px | D07V056Once | 99223 | initial hospital care |  |
| Outpatient CPT Px | D07V398Once | 99222 | initial hospital care |  |
| Outpatient CPT Px | D07V685Once | 93545 | inject for coronary x-rays |  |
| Outpatient CPT Px | D07V180Freq | 99254 | inpatient consultation |  |
| Outpatient CPT Px | D07V180Once | 99254 | inpatient consultation |  |
| Outpatient CPT Px | D07V251Freq | 99253 | inpatient consultation |  |
| Outpatient CPT Px | D07V251Once | 99253 | inpatient consultation |  |
| Outpatient CPT Px | D07V500Freq | 99255 | inpatient consultation |  |
| Outpatient CPT Px | D07V500Once | 99255 | inpatient consultation |  |
| Outpatient CPT Px | D07V112Once | 83550 | iron binding test |  |
| Outpatient CPT Px | D07V283Once | 83615 | lactate (ld) (ldh) enzyme | Y |
| Outpatient CPT Px | D07V003Spor | 80061 | lipid panel | Y |
| Outpatient CPT Px | D07V001Once | 80048 | metabolic panel total ca |  |
| Outpatient CPT Px | D07V142Once | 82043 | microalbumin, quantitative |  |
| Outpatient CPT Px | D07V030Once | 87186 | microbe susceptible, mic |  |
| Outpatient CPT Px | D07V209Once | 82270 | occulture blood, feces | Y |
| Outpatient CPT Px | D07V092Freq | 99212 | office/outpatient visit, est | Y |
| Outpatient CPT Px | D07V092Spor | 99212 | office/outpatient visit, est | Y |
| Outpatient CPT Px | D07V215Freq | 99211 | office/outpatient visit, est |  |
| Outpatient CPT Px | D07V644Once | 92135 | ophth dx imaging post seg |  |
| Outpatient CPT Px | D07V143Freq | 85610 | prothrombin time |  |
| Outpatient CPT Px | D07V482Once | 90801 | psych dx interview |  |
| Outpatient CPT Px | D07V241Freq | 93042 | rhythm ecg, report |  |
| Outpatient CPT Px | D07V241Once | 93042 | rhythm ecg, report |  |
| Outpatient CPT Px | D07V000Freq | 36415 | routine venipuncture | Y |
| Outpatient CPT Px | D07V000Spor | 36415 | routine venipuncture | Y |
| Outpatient CPT Px | D07V057Freq | 99233 | subsequent hospital care |  |
| Outpatient CPT Px | D07V057Once | 99233 | subsequent hospital care |  |
| Outpatient CPT Px | D07V057Spor | 99233 | subsequent hospital care |  |
| Outpatient CPT Px | D07V176Freq | 99231 | subsequent hospital care |  |
| Outpatient CPT Px | D07V176Once | 99231 | subsequent hospital care |  |
| Outpatient CPT Px | D07V176Spor | 99231 | subsequent hospital care |  |
| Outpatient CPT Px | D07V399Freq | 99232 | subsequent hospital care |  |
| Outpatient CPT Px | D07V399Once | 99232 | subsequent hospital care |  |
| Outpatient CPT Px | D07V399Spor | 99232 | subsequent hospital care |  |
| Outpatient CPT Px | D07V226Freq | 81001 | urinalysis, auto w/scope |  |
| Outpatient CPT Px | D07V226Once | 81001 | urinalysis, auto w/scope | Y |
| Outpatient CPT Px | D07V005Freq | 81000 | urinalysis, nonauto w/scope | Y |
| Outpatient CPT Px | D07V105Once | 82607 | vitamin b-12 | Y |
| Outpatient CPT Px | D07V737Freq | X0030 | ambulance service, basic life support |  |
| Outpatient CPT Px | D07V737Once | X0030 | ambulance service, basic life support |  |
| Outpatient CPT Px | D07V738Freq | X0034 | ambulance, per mile transport |  |
| Outpatient CPT Px | D07V738Once | X0034 | ambulance, per mile transport |  |
| Outpatient CPT Px | D07V079Once | 74000 | x-ray abdomen |  |
| Outpatient CPT Px | D07V196Once | Z2932 | [local codes] |  |
| Outpatient CPT Px | D07V137Once | Z3136 |  |  |
| Outpatient CPT Px | D07V073Once | Z9525 |  |  |
| Outpatient CPT Px | D07V073Spor | Z9525 |  |  |
| Outpatient ICD-9 Dx | D05V161Freq | 793 | abn find-body struct nos |  |
| Outpatient ICD-9 Dx | D05V161Once | 793 | abn find-body struct nos |  |
| Outpatient ICD-9 Dx | D05V023Freq | 790 | abnormal blood findings |  |
| Outpatient ICD-9 Dx | D05V023Once | 790 | abnormal blood findings |  |
| Outpatient ICD-9 Dx | D05V117Once | 794 | abnormal function study |  |
| Outpatient ICD-9 Dx | D05V300Freq | 410 | acute myocardial infarct |  |
| Outpatient ICD-9 Dx | D05V300Once | 410 | acute myocardial infarct |  |
| Outpatient ICD-9 Dx | D05V300Spor | 410 | acute myocardial infarct |  |
| Outpatient ICD-9 Dx | D05V251Freq | 584 | acute renal failure |  |
| Outpatient ICD-9 Dx | D05V251Once | 584 | acute renal failure |  |
| Outpatient ICD-9 Dx | D05V251Spor | 584 | acute renal failure |  |
| Outpatient ICD-9 Dx | D05V057Freq | 285 | anemia nec/nos |  |
| Outpatient ICD-9 Dx | D05V057Once | 285 | anemia nec/nos |  |
| Outpatient ICD-9 Dx | D05V188Freq | 413 | angina pectoris |  |
| Outpatient ICD-9 Dx | D05V188Once | 413 | angina pectoris |  |
| Outpatient ICD-9 Dx | D05V046Freq | 440 | atherosclerosis |  |
| Outpatient ICD-9 Dx | D05V046Once | 440 | atherosclerosis |  |
| Outpatient ICD-9 Dx | D05V018Freq | 041 | bact inf in oth dis/nos |  |
| Outpatient ICD-9 Dx | D05V018Once | 041 | bact inf in oth dis/nos |  |
| Outpatient ICD-9 Dx | D05V157Freq | 427 | cardiac dysrhythmias |  |
| Outpatient ICD-9 Dx | D05V157Once | 427 | cardiac dysrhythmias |  |
| Outpatient ICD-9 Dx | D05V135Freq | 425 | cardiomyopathy |  |
| Outpatient ICD-9 Dx | D05V135Once | 425 | cardiomyopathy |  |
| Outpatient ICD-9 Dx | D05V060Once | 785 | cardiovascular sys symp |  |
| Outpatient ICD-9 Dx | D05V040Freq | 366 | cataract |  |
| Outpatient ICD-9 Dx | D05V040Once | 366 | cataract |  |
| Outpatient ICD-9 Dx | D05V085Once | 681 | cellulitis |  |
| Outpatient ICD-9 Dx | D05V119Freq | 496 | chr airway obstruct nec |  |
| Outpatient ICD-9 Dx | D05V119Once | 496 | chr airway obstruct nec |  |
| Outpatient ICD-9 Dx | D05V119Spor | 496 | chr airway obstruct nec |  |
| Outpatient ICD-9 Dx | D05V120Freq | 491 | chronic bronchitis |  |
| Outpatient ICD-9 Dx | D05V120Once | 491 | chronic bronchitis |  |
| Outpatient ICD-9 Dx | D05V120Spor | 491 | chronic bronchitis |  |
| Outpatient ICD-9 Dx | D05V172Freq | 585 | chronic renal failure |  |
| Outpatient ICD-9 Dx | D05V172Once | 585 | chronic renal failure |  |
| Outpatient ICD-9 Dx | D05V172Spor | 585 | chronic renal failure |  |
| Outpatient ICD-9 Dx | D05V031Freq | 707 | chronic ulcer of skin |  |
| Outpatient ICD-9 Dx | D05V031Once | 707 | chronic ulcer of skin |  |
| Outpatient ICD-9 Dx | D05V031Spor | 707 | chronic ulcer of skin |  |
| Outpatient ICD-9 Dx | D05V133Once | 426 | conduction disorders |  |
| Outpatient ICD-9 Dx | D05V207Freq | 436 | cva |  |
| Outpatient ICD-9 Dx | D05V207Once | 436 | cva |  |
| Outpatient ICD-9 Dx | D05V207Spor | 436 | cva |  |
| Outpatient ICD-9 Dx | D05V076Freq | 110 | dermatophytosis |  |
| Outpatient ICD-9 Dx | D05V076Once | 110 | dermatophytosis |  |
| Outpatient ICD-9 Dx | D05V076Spor | 110 | dermatophytosis |  |
| Outpatient ICD-9 Dx | D05V000Freq | 250 | diabetes mellitus |  |
| Outpatient ICD-9 Dx | D05V000Once | 250 | diabetes mellitus |  |
| Outpatient ICD-9 Dx | D05V000Spor | 250 | diabetes mellitus |  |
| Outpatient ICD-9 Dx | D05V149Freq | 275 | dis mineral metabolism |  |
| Outpatient ICD-9 Dx | D05V149Spor | 275 | dis mineral metabolism |  |
| Outpatient ICD-9 Dx | D05V038Freq | 703 | diseases of nail |  |
| Outpatient ICD-9 Dx | D05V038Once | 703 | diseases of nail |  |
| Outpatient ICD-9 Dx | D05V038Spor | 703 | diseases of nail |  |
| Outpatient ICD-9 Dx | D05V140Freq | V58 | encountr proc/aftrcr nec |  |
| Outpatient ICD-9 Dx | D05V140Once | V58 | encountr proc/aftrcr nec |  |
| Outpatient ICD-9 Dx | D05V140Spor | V58 | encountr proc/aftrcr nec |  |
| Outpatient ICD-9 Dx | D05V003Freq | 401 | essential hypertension |  |
| Outpatient ICD-9 Dx | D05V003Once | 401 | essential hypertension |  |
| Outpatient ICD-9 Dx | D05V003Spor | 401 | essential hypertension |  |
| Outpatient ICD-9 Dx | D05V021Freq | 276 | fluid/electrolyte dis |  |
| Outpatient ICD-9 Dx | D05V021Once | 276 | fluid/electrolyte dis |  |
| Outpatient ICD-9 Dx | D05V021Spor | 276 | fluid/electrolyte dis |  |
| Outpatient ICD-9 Dx | D05V220Freq | 578 | gastrointestinal hemorr |  |
| Outpatient ICD-9 Dx | D05V220Once | 578 | gastrointestinal hemorr |  |
| Outpatient ICD-9 Dx | D05V205Once | V70 | general medical exam |  |
| Outpatient ICD-9 Dx | D05V013Once | 780 | general symptoms |  |
| Outpatient ICD-9 Dx | D05V180Freq | 428 | heart failure |  |
| Outpatient ICD-9 Dx | D05V180Once | 428 | heart failure |  |
| Outpatient ICD-9 Dx | D05V180Spor | 428 | heart failure |  |
| Outpatient ICD-9 Dx | D05V274Once | V12 | hx of disease nec |  |
| Outpatient ICD-9 Dx | D05V156Once | 402 | hypertensive heart dis |  |
| Outpatient ICD-9 Dx | D05V156Spor | 402 | hypertensive heart dis |  |
| Outpatient ICD-9 Dx | D05V170Freq | 403 | hypertensive renal dis |  |
| Outpatient ICD-9 Dx | D05V170Once | 403 | hypertensive renal dis |  |
| Outpatient ICD-9 Dx | D05V170Spor | 403 | hypertensive renal dis |  |
| Outpatient ICD-9 Dx | D05V185Freq | 429 | ill-defined heart dis |  |
| Outpatient ICD-9 Dx | D05V185Once | 429 | ill-defined heart dis |  |
| Outpatient ICD-9 Dx | D05V193Freq | 280 | iron deficiency anemias |  |
| Outpatient ICD-9 Dx | D05V193Once | 280 | iron deficiency anemias |  |
| Outpatient ICD-9 Dx | D05V193Spor | 280 | iron deficiency anemias |  |
| Outpatient ICD-9 Dx | D05V122Once | 305 | nondependent drug abuse |  |
| Outpatient ICD-9 Dx | D05V224Once | 783 | nutrit/metab/devel symp |  |
| Outpatient ICD-9 Dx | D05V029Once | V71 | observation-suspect cond |  |
| Outpatient ICD-9 Dx | D05V132Freq | 411 | oth ac ischemic hrt dis |  |
| Outpatient ICD-9 Dx | D05V132Once | 411 | oth ac ischemic hrt dis |  |
| Outpatient ICD-9 Dx | D05V131Freq | 414 | oth chr ischemic hrt dis |  |
| Outpatient ICD-9 Dx | D05V131Once | 414 | oth chr ischemic hrt dis |  |
| Outpatient ICD-9 Dx | D05V131Spor | 414 | oth chr ischemic hrt dis |  |
| Outpatient ICD-9 Dx | D05V110Once | 459 | oth circulatory disease |  |
| Outpatient ICD-9 Dx | D05V126Freq | 424 | oth endocardial disease |  |
| Outpatient ICD-9 Dx | D05V126Once | 424 | oth endocardial disease |  |
| Outpatient ICD-9 Dx | D05V268Once | V15 | oth hx of health hazards |  |
| Outpatient ICD-9 Dx | D05V290Freq | 799 | oth ill-def morbid/mortl | Y |
| Outpatient ICD-9 Dx | D05V290Once | 799 | oth ill-def morbid/mortl | Y |
| Outpatient ICD-9 Dx | D05V290Spor | 799 | oth ill-def morbid/mortl | Y |
| Outpatient ICD-9 Dx | D05V234Once | 251 | oth pancreatic disorder |  |
| Outpatient ICD-9 Dx | D05V165Freq | 443 | oth periph vascular dis |  |
| Outpatient ICD-9 Dx | D05V165Once | 443 | oth periph vascular dis |  |
| Outpatient ICD-9 Dx | D05V020Freq | 593 | oth renal & ureteral dis |  |
| Outpatient ICD-9 Dx | D05V020Once | 593 | oth renal & ureteral dis |  |
| Outpatient ICD-9 Dx | D05V227Freq | 746 | other congen heart anom |  |
| Outpatient ICD-9 Dx | D05V227Once | 746 | other congen heart anom |  |
| Outpatient ICD-9 Dx | D05V095Freq | 518 | other lung diseases |  |
| Outpatient ICD-9 Dx | D05V095Once | 518 | other lung diseases |  |
| Outpatient ICD-9 Dx | D05V039Once | 729 | other soft tissue dis |  |
| Outpatient ICD-9 Dx | D05V094Once | 726 | periph enthesopathies |  |
| Outpatient ICD-9 Dx | D05V026Freq | 511 | pleurisy |  |
| Outpatient ICD-9 Dx | D05V026Once | 511 | pleurisy |  |
| Outpatient ICD-9 Dx | D05V216Freq | 486 | pneumonia |  |
| Outpatient ICD-9 Dx | D05V216Once | 486 | pneumonia |  |
| Outpatient ICD-9 Dx | D05V216Spor | 486 | pneumonia |  |
| Outpatient ICD-9 Dx | D05V340Freq | 514 | pulm congest/hypostasis |  |
| Outpatient ICD-9 Dx | D05V340Once | 514 | pulm congest/hypostasis |  |
| Outpatient ICD-9 Dx | D05V167Freq | 586 | renal failure nos |  |
| Outpatient ICD-9 Dx | D05V167Once | 586 | renal failure nos |  |
| Outpatient ICD-9 Dx | D05V167Spor | 586 | renal failure nos |  |
| Outpatient ICD-9 Dx | D05V342Freq | 996 | replace & graft complic |  |
| Outpatient ICD-9 Dx | D05V342Once | 996 | replace & graft complic |  |
| Outpatient ICD-9 Dx | D05V342Spor | 996 | replace & graft complic |  |
| Outpatient ICD-9 Dx | D05V022Freq | 786 | resp sys/oth chest symp |  |
| Outpatient ICD-9 Dx | D05V022Once | 786 | resp sys/oth chest symp |  |
| Outpatient ICD-9 Dx | D05V022Spor | 786 | resp sys/oth chest symp |  |
| Outpatient ICD-9 Dx | D05V050Freq | 362 | retinal disorders nec |  |
| Outpatient ICD-9 Dx | D05V050Once | 362 | retinal disorders nec |  |
| Outpatient ICD-9 Dx | D05V050Spor | 362 | retinal disorders nec |  |
| Outpatient ICD-9 Dx | D05V065Once | 295 | schizophrenic disorders |  |
| Outpatient ICD-9 Dx | D05V065Spor | 295 | schizophrenic disorders |  |
| Outpatient ICD-9 Dx | D05V035Freq | 038 | septicemia |  |
| Outpatient ICD-9 Dx | D05V035Once | 038 | septicemia |  |
| Outpatient ICD-9 Dx | D05V035Spor | 038 | septicemia |  |
| Outpatient ICD-9 Dx | D05V036Freq | V72 | special examinations | Y |
| Outpatient ICD-9 Dx | D05V036Once | V72 | special examinations | Y |
| Outpatient ICD-9 Dx | D05V111Freq | 451 | thrombophlebitis |  |
| Outpatient ICD-9 Dx | D05V111Once | 451 | thrombophlebitis |  |
| Outpatient ICD-9 Dx | D05V051Once | 435 | transient cereb ischemia |  |
| Outpatient ICD-9 Dx | D05V041Once | V04 | vaccin for viral disease |  |
| Outpatient ICD-9 Dx | D05V090Once | 454 | varicose veins |  |
| Outpatient ICD-9 Px | D06V084Freq | 95 | eye & ear dx/treatment |  |
| Outpatient ICD-9 Px | D06V017Once | 89 | interview/consult/exam | Y |
| Outpatient ICD-9 Px | D06V002Once | 88 | other dx radiology |  |
| Outpatient ICD-9 Px | D06V002Spor | 88 | other dx radiology |  |
| Outpatient ICD-9 Px | D06V071Once | 84 | other musculoskelet proc |  |
| Outpatient ICD-9 Px | D06V010Once | 99 | other nonoperative proc |  |
| Outpatient ICD-9 Px | D06V022Freq | 39 | other ops on vessels |  |
| Outpatient ICD-9 Px | D06V022Once | 39 | other ops on vessels |  |
| Outpatient ICD-9 Px | D06V000Once | 86 | skin & subq operations |  |
| Outpatient ICD-9 Px | D06V036Once | 38 | vessel inc/excis/occlus |  |

CPT = Current Procedural Terminology; Dx = diagnosis; ICD-9 = International Classification of Diseases, 9th Revision; NDC = National Drug Code; Px = procedure

* in a prespecified secondary analysis, variables with z-bias = 5 were excluded from the propensity score as they were strongly related to exposure, but not outcome

Prespecified covariates are detailed in **Additional file 1. Table S1**.

**Additional file 1.** **Figure S1. Standardized differences for baseline characteristics of thiazolidinedione users, before vs. after high-dimensional propensity score matching**

**Additional file 1.** **Figure S2. Kaplan-Meier curve depicting the probability of sudden cardiac arrest / ventricular arrhythmia upon new use of rosiglitazone vs. pioglitazone, in all users (N = 500,091)**

Solid line is pioglitazone. Dashed line is rosiglitazone. P-value for log-rank test = 0.09.

**Additional file 1.** **Figure S3. Propensity score distributions pre- vs. post-matching**

Panel A: Pre-matching


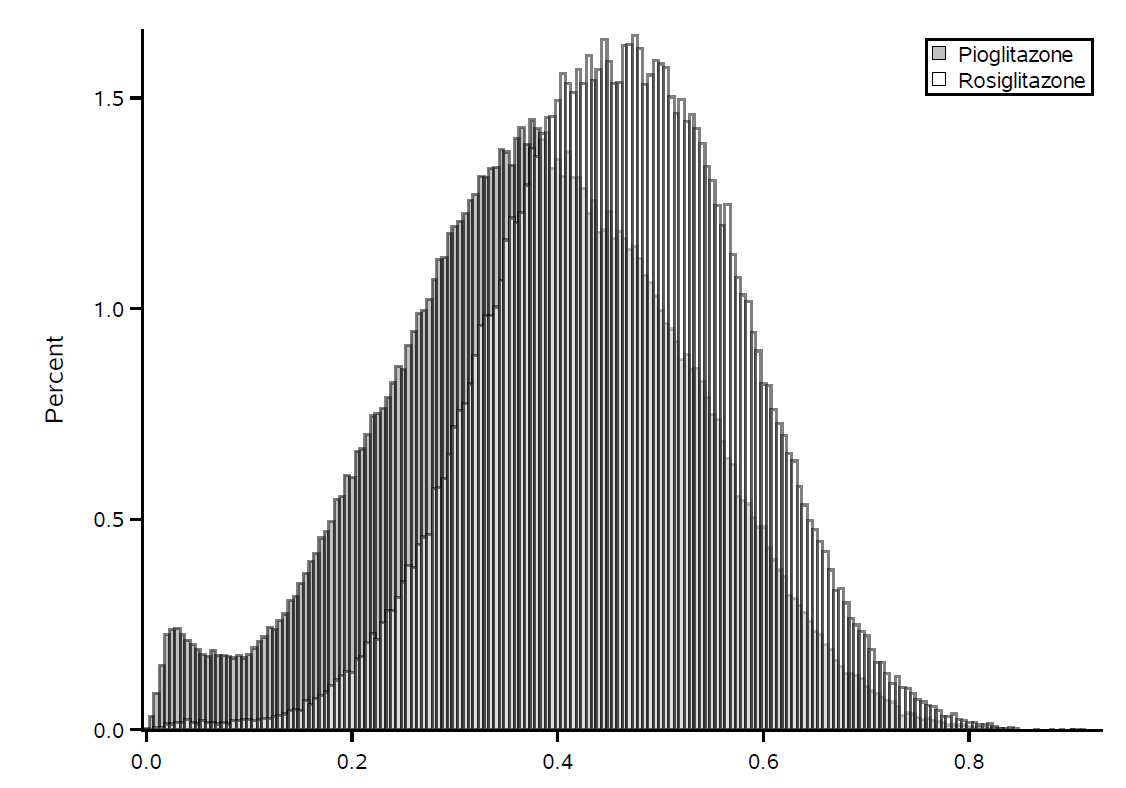


Panel B: Post-matching


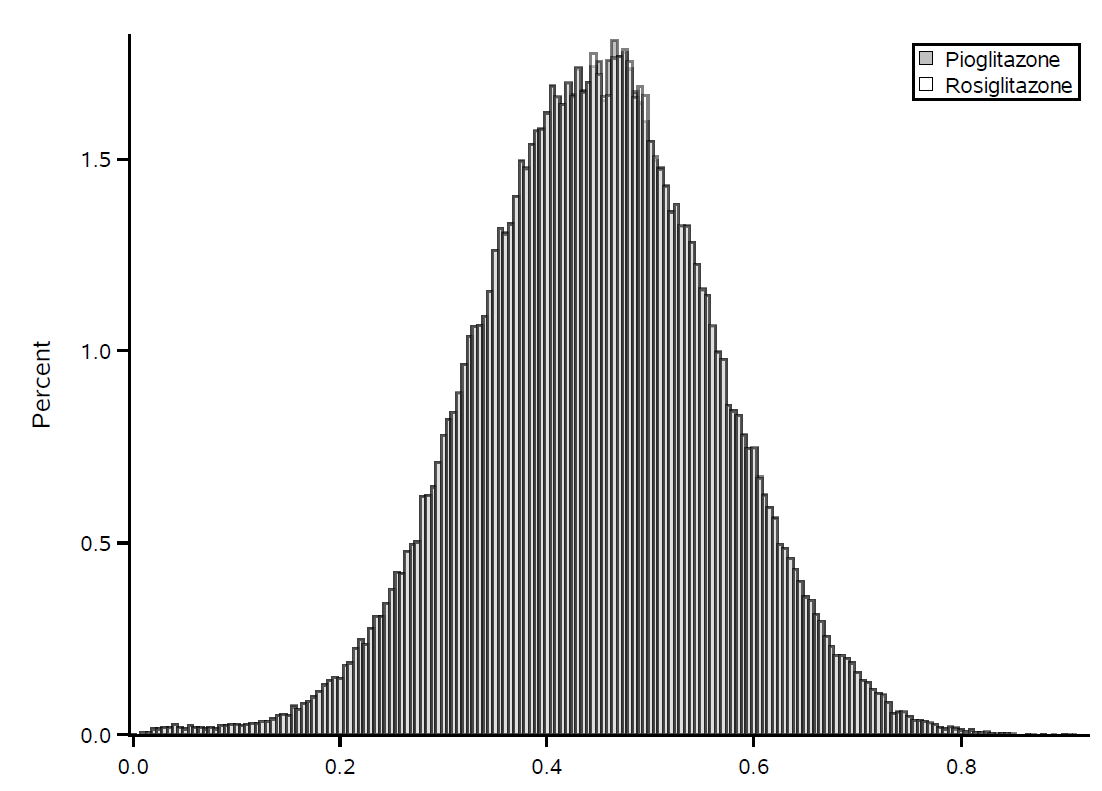


X-axis values are propensity scores [range: 0.0 to 1.0].

**Additional file 1.** **Figure S4. Confounder-adjusted hazard ratios for associations between thiazolidinediones and sudden cardiac arrest / ventricular arrhythmia, by average daily dose | Medicaid**

Panel A: pioglitazone (N = 293,519)

Panel B: rosiglitazone (N = 205,266)

HR = hazard ratio; ref = referent

Squares depict hazard ratios for the primary outcome of sudden cardiac arrest and ventricular arrhythmia.

**Additional file 1.** **Figure S5. Confounder-adjusted hazard ratios for associations between thiazolidinediones and sudden cardiac arrest / ventricular arrhythmia, by average daily dose | Optum**

Panel A: pioglitazone (N = 189,935)

Panel B: rosiglitazone (N = 103,658)

HR = hazard ratio; ref = referent

Squares depict hazard ratios for the primary outcome of sudden cardiac arrest and ventricular arrhythmia.
